# Comparison of bias and resolvability in single-cell and single-transcript methods

**DOI:** 10.1038/s42003-021-02138-6

**Published:** 2021-06-02

**Authors:** Jayan Rammohan, Steven P. Lund, Nina Alperovich, Vanya Paralanov, Elizabeth A. Strychalski, David Ross

**Affiliations:** National Institute of Standards and Technology, Gaithersburg, MD USA

**Keywords:** Single-molecule biophysics, Bacterial transcription, Single-cell imaging, Gene expression analysis

## Abstract

Single-cell and single-transcript measurement methods have elevated our ability to understand and engineer biological systems. However, defining and comparing performance between methods remains a challenge, in part due to the confounding effects of experimental variability. Here, we propose a generalizable framework for performing multiple methods in parallel using split samples, so that experimental variability is shared between methods. We demonstrate the utility of this framework by performing 12 different methods in parallel to measure the same underlying reference system for cellular response. We compare method performance using quantitative evaluations of bias and resolvability. We attribute differences in method performance to steps along the measurement process such as sample preparation, signal detection, and choice of measurand. Finally, we demonstrate how this framework can be used to benchmark different methods for single-transcript detection. The framework we present here provides a practical way to compare performance of any methods.

## Abstract (web summary)

Rammohan et al. propose a generalizable framework for performing multiple methods in parallel using split samples, so that experimental variability is defined and compared between methods. Their framework provides a practical solution for benchmarking and comparing the performance of any method, illustrated by analysing single-cell and single-transcript methods.

## Introduction

Single-cell^1–3^ and single-transcript^4–8^ methods for measurements of gene expression are revolutionizing our ability to understand and engineer biological systems^9–11^, but evaluation of method performance remains a challenge^12,13^. Bias and resolvability are two important, practical aspects of single-cell and single-transcript method performance. Relative bias, i.e., systematic measurement differences between methods, can influence the conclusions made about cellular response^3,14^. Resolvability, i.e., the ability to resolve different levels of gene expression, can impact cell-sorting^15–17^, sensor engineering^18^, and analysis of differential gene expression^2^. Comparison of bias and resolvability between methods is challenging because experimental variability can be introduced at any step in the measurement process, including cell culture, sample preparation, signal detection, and choice of measurand. Therefore, a rigorous comparison of method performance would benefit from an experiment design framework that mitigates experimental variability introduced from each of these steps.

Here, we present Bias and Resolvability Attribution using Split Samples (BRASS), a framework for quantitative evaluation and comparison of methods. In this framework, multiple single-cell and single-transcript methods are performed in parallel on cells harvested from the same original culture. Harvested cells are then divided (split) at each step along the measurement process: first for different sample preparations, next for different signal detections, and finally for different measurands. Consequently, the impact of experimental variability is mitigated, because sources of experimental variability are shared by different methods whenever possible. We demonstrate the utility of BRASS by performing a total of 12 different methods in parallel, all measuring the same underlying reference system for cellular response^19^. To gauge the impact of relative bias between methods, we fit measurements from different methods with the same model of cellular response, and compare the resulting parameters estimated from each method. To evaluate the resolvability of gene expression for each method, we use a quantitative metric to calculate the extent and direction of overlap between distributions measured at different levels of gene expression. Furthermore, using pairwise comparisons between measurements, we systematically attribute differences in measurement performance to steps of the measurement process including sample preparation, signal detection, and choice of measurand. Finally, we show how this split-sample approach can be used to benchmark a versatile method for single-transcript detection in bacteria (hybridization chain reaction, HCR)^20^ against a traditional technique (fluorescence in situ hybridization, FISH)^5,21^.

## Results

### Method comparison using split-sample measurements in parallel

**Fig. 1:**
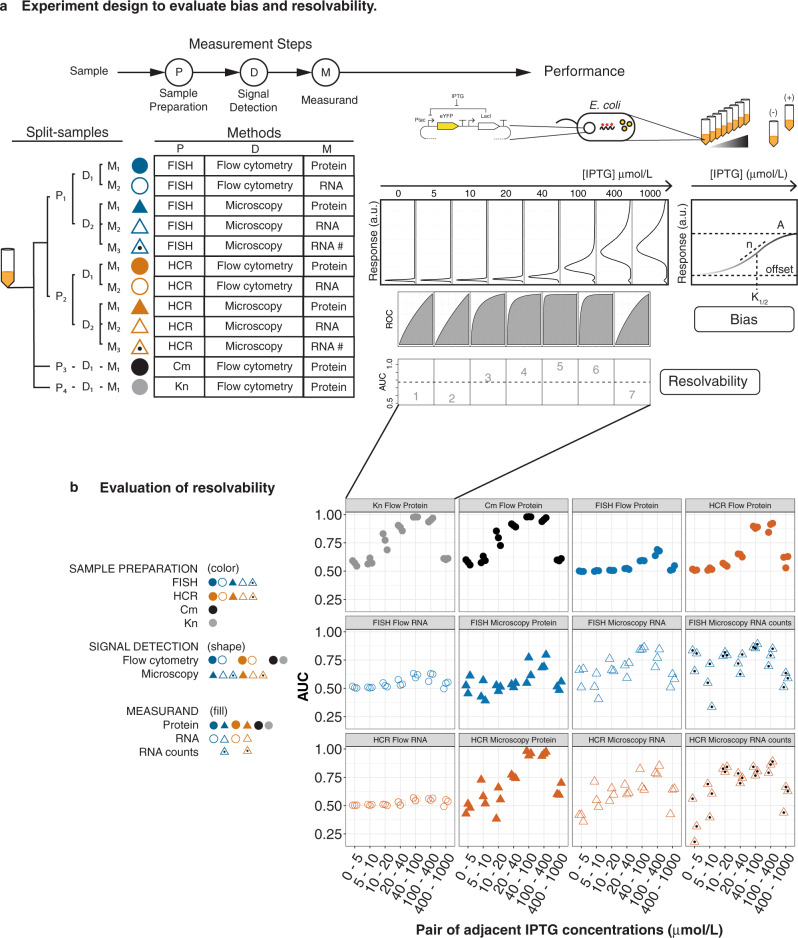
Experiment design and evaluation of resolvability. **a** In this study, each cell culture is divided (split) to perform multiple methods in parallel for measuring the same underlying system of cellular sense and response. Eight cultures were measured over a range of induction with IPTG. Methods include various combinations of sample preparation, signal detection, and choice of measurand. Resolvability is quantitatively assessed using Area Under the receiver operator characteristic Curve (AUC) calculated across a range of IPTG concentrations. Relative bias is assessed by modeling cellular response using measurements from each method and comparing the resulting parameters. **b** Evaluation of resolvability with AUC profiles for all 12 methods. In each panel, the plotted symbols show the AUC for pairs of adjacent IPTG concentrations as indicated on the *x*-axis.

To compare the performance of different methods, we designed a readily adoptable framework in which multiple methods are applied to sequentially split samples (Fig. 1a and Supplementary Note 1). At each step in the measurement process, the original sample is split: first for sample preparation, next for signal detection, and finally for choice of measurand (Fig. 1a). This design is ideal for comparing methods: with split samples, differences in method performance can be distinguished from replicate-to-replicate variability at each step in the measurement process. Furthermore, because this framework can be used to attribute differences between methods to particular steps in the measurement process, it is ideal for understanding how measurement steps contribute to overall method performance.

**Fig. 2:**
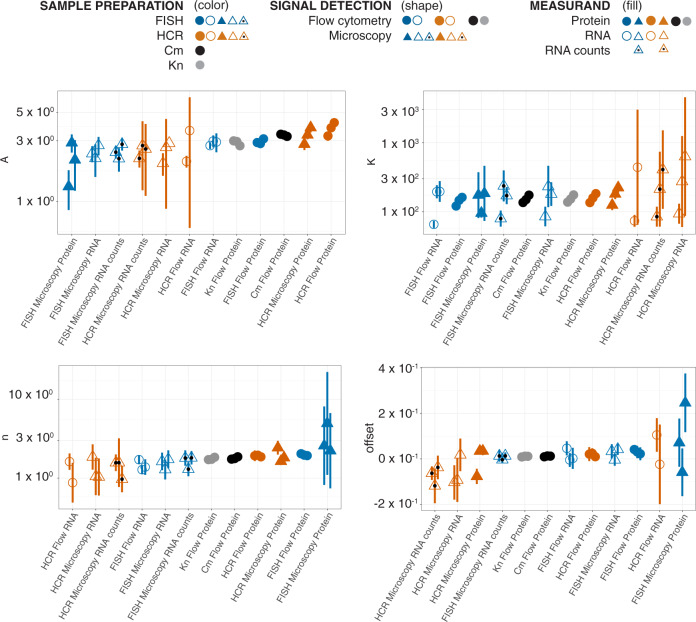
Evaluation of relative bias in Hill parameters. Hill parameters for fits to RPU-normalized data are shown for all 12 methods. Symbols represent parameter estimates for methods according to legend. Error bars indicate 95% confidence intervals from nonlinear least-squares fits. For each parameter, methods are ordered on *x*-axis from left-to-right from lowest to highest average parameters value.

**Fig. 3:**
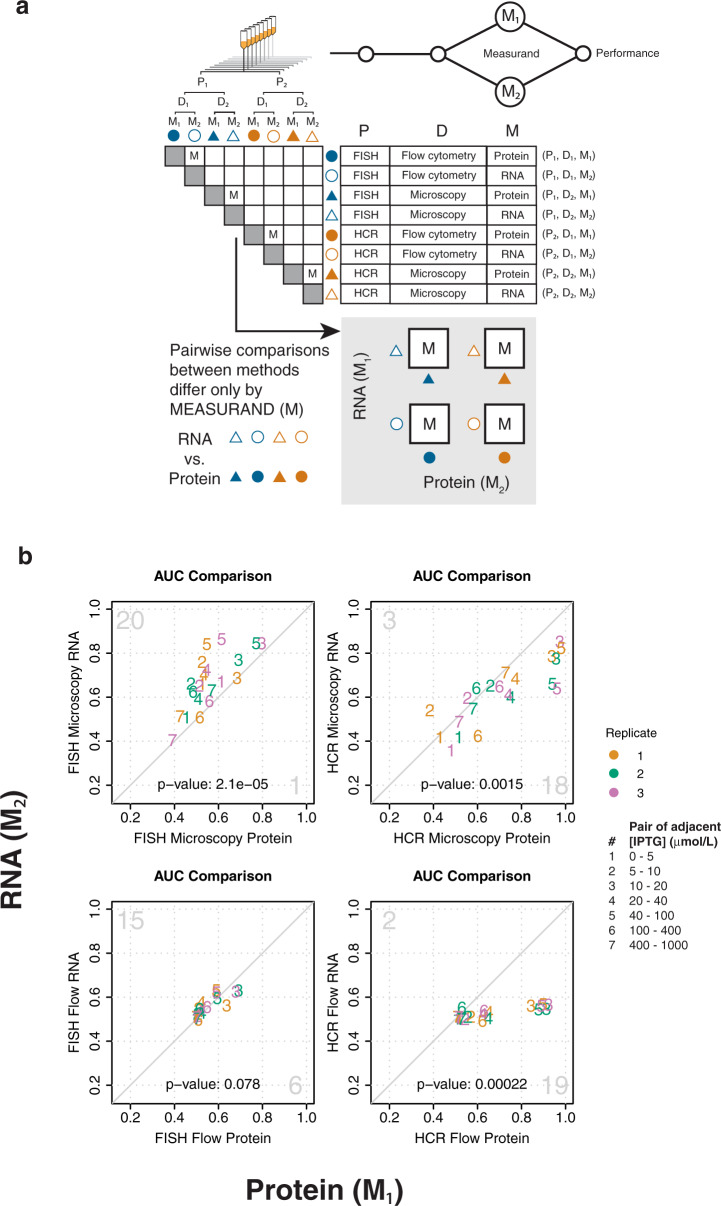
Method performance can be attributed to measurand. **a** Pairwise comparisons of methods that share the same steps for sample preparation and signal detection, but differ in measurand (“M” squares in matrix), are used to attribute measurement performance to measurand. The four boxes containing “M” under the matrix represent these same pairwise comparisons. **b** Pairwise AUC plots of measurements in (**a**) are used to compare resolvability between RNA and protein measurands. Diagonal line indicates equivalent resolvability between the two methods. Pairs of adjacent IPTG concentrations are shown as numbers within the plots, as indicated in the figure legend. Color indicates biological replicates one (orange), two (green), and three (purple), as indicated in the figure legend. Large gray numbers in the top-left and bottom right-corners indicate how many AUC’s were higher for the method plotted on the *y*-axis or *x*-axis, respectively. The two-sided *p*-values for a sign test are shown within each plot.

**Fig. 4:**
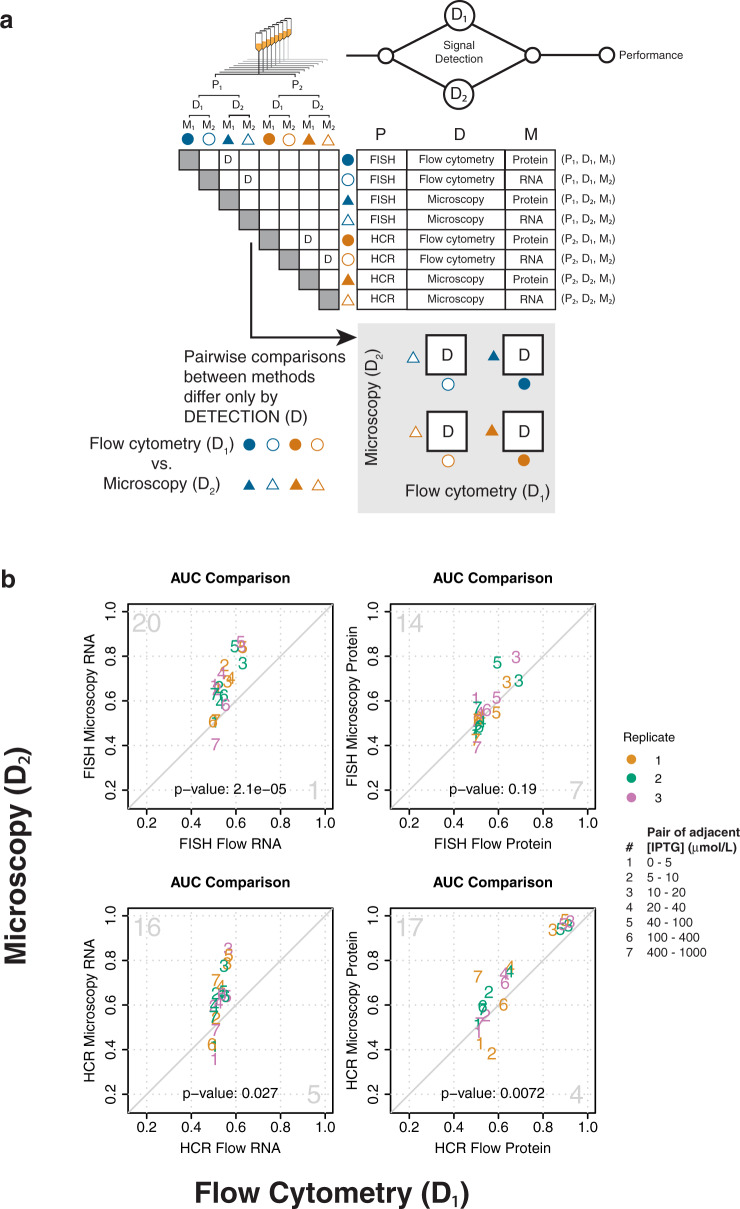
Method performance can be attributed to signal detection. **a** Pairwise comparisons of methods that share the same steps for sample preparation and measurand, but differ in signal detection (indicated by “D” in the matrix), are used to attribute measurement performance to signal detection. The four boxes containing “D” under the matrix represent these same pairwise comparisons. **b** Pairwise AUC plots of measurements in (**a**) to compare resolvability between flow cytometry and microscopy. Diagonal line indicates equivalent resolvability between the two methods. Pairs of adjacent IPTG concentrations are shown as numbers within the plots, as indicated in the figure legend. Color indicates biological replicates one (orange), two (green), and three (purple), as indicated in the figure legend. Large gray numbers in the top-left and bottom right-corners indicate how many AUC’s were higher for the method plotted on the *y*-axis or *x*-axis, respectively. The two-sided *p*-values for a sign test are shown within each plot.

**Fig. 5:**
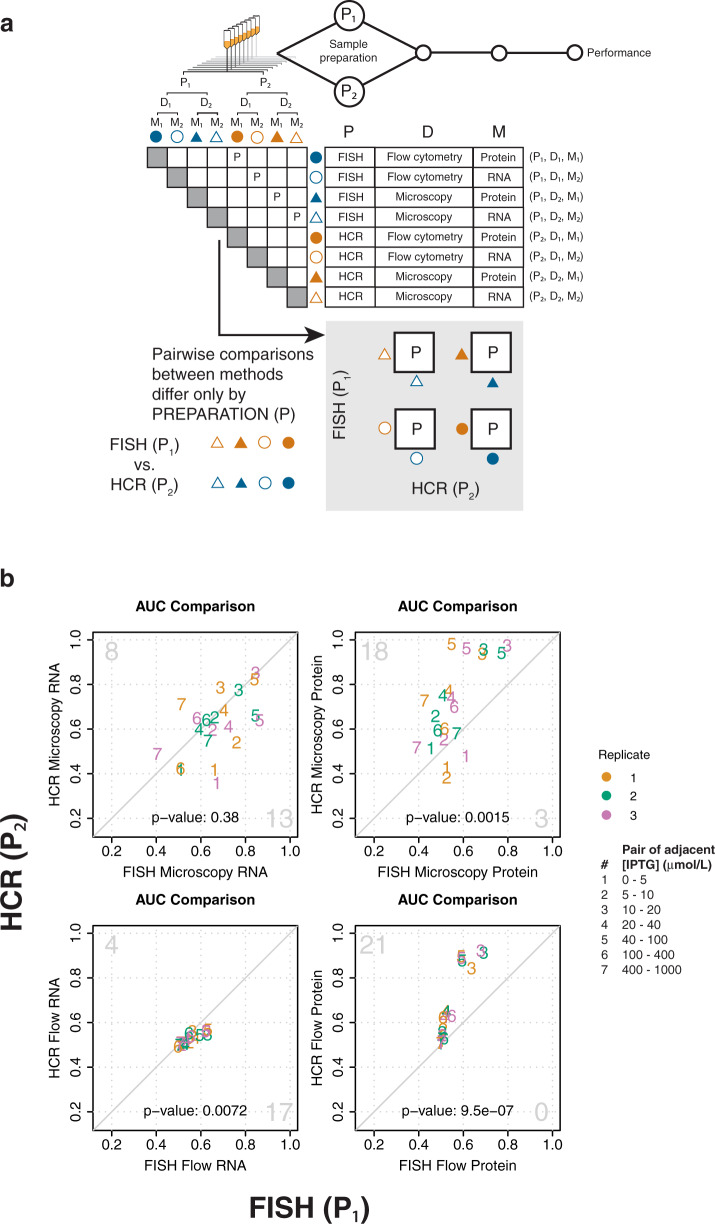
Method performance can be attributed to sample preparation. **a** Pairwise comparisons of methods that share the same steps for signal detection and measurand, but differ in sample preparation (indicated by “P” in the matrix), are used to attribute measurement performance to sample preparation. The four boxes containing “P” under the matrix represent these same pairwise comparisons. **b** Pairwise AUC plots of measurements in (**a**) are used to compare resolvability between sample preparation methods (FISH versus HCR). Diagonal line indicates equivalent resolvability between the two methods. Pairs of adjacent IPTG concentrations are shown as numbers within the plots, as indicated in the figure legend. Color indicates biological replicates one (orange), two (green), and three (purple), as indicated in the figure legend. Large gray numbers in the top-left and bottom right-corners indicate how many AUC’s were higher for the method plotted on the *y*-axis or *x*-axis, respectively. The two-sided *p*-values for a sign test are shown within each plot.

**Fig. 6:**
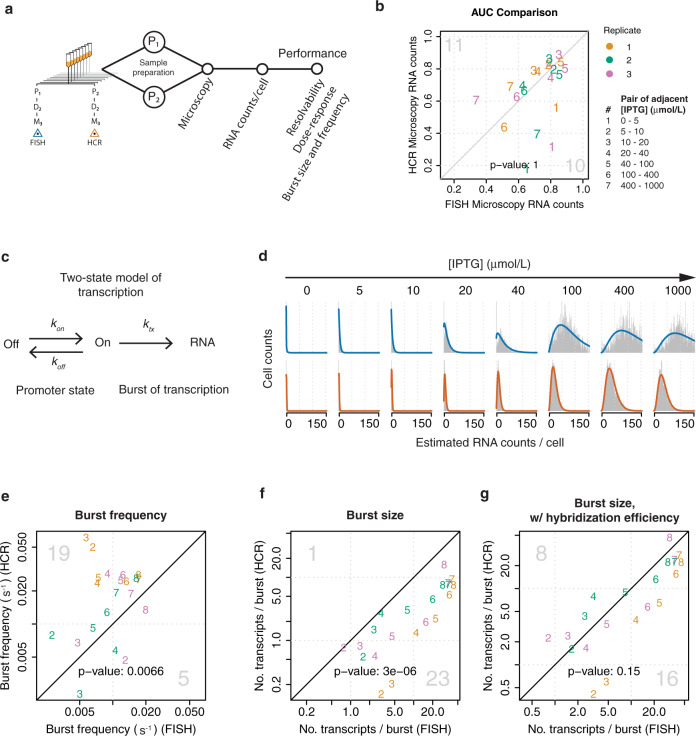
Performance of single-transcript methods can be attributed to RNA labeling strategy. **a** Performance of single-transcript methods was compared using cells that shared the same steps of the measurement process except for the RNA labeling step. **b** Resolvability of FISH and HCR was assessed by plotting AUC calculated from adjacent stimulus levels. Pairs of adjacent IPTG concentrations are shown as numbers within the plots, as indicated in figure legend. **c** A two-state promoter model was used to evaluate transcription kinetics. **d** Negative binomials were used to fit single-transcript distributions for FISH (blue) and HCR (dark orange). **e** Estimates of burst frequency are plotted for FISH versus HCR. The RNA lifetime was assumed to be a constant (2.8 min). **f** Estimates of burst size, and **g** estimates of burst size after correcting for hybridization efficiency, are plotted for FISH versus HCR. For parts (**f**) and (**g**), burst size is the number of transcripts per burst. For parts (**e**), (**f**), and (**g**), the scatter plot numbers 2, 3, 4, 5, 6, 7, and 8 represent 5, 10, 20, 40, 100, 400, and 1000 μmol/L IPTG, respectively. The 0 μmol/L IPTG case is not shown, in order to more easily see the trend for the remaining induction conditions. For (**b**), (**e**), (**f**), and (**g**): Diagonal line indicates equivalent performance between the two methods. Color indicates biological replicates one (orange), two (green), and three (purple), as indicated in the figure legend. Large gray numbers in the top-left and bottom right-corners indicate how many values were higher for the method plotted on the *y*-axis or *x*-axis, respectively. The two-sided *p*-values for a sign test are shown within each plot.

To demonstrate the utility of BRASS, we compared different methods for measuring the same underlying model system for cellular sense and response, previously used as a reference in automated genetic circuit design^19^: regulation of gene expression in *Escherichia coli* (*E. coli*) by the lac repressor, and induction with isopropyl-β-D-thiogalactopyranoside (IPTG) (Fig. 1a and Supplementary Fig. 1). We grew ten different cultures representing different levels of the expected response. Eight cultures contained *E. coli* with an expression of enhanced yellow fluorescent protein (eYFP) controlled by an IPTG-inducible plasmid. One culture contained *E. coli* with a positive-control plasmid expressing eYFP at a fixed level from the J23101 promoter, which has previously been used as a living reference for normalization to Relative Promoter Units (RPU)^22,23^. The last culture contained *E. coli* with a negative-control plasmid lacking eYFP. We harvested cells from each culture at mid-log phase and sequentially split the cell samples for sample preparation (P in Figs. 1–6), signal detection (D in Figs. 1–5), and measurand (M in Figs. 1–5). This resulted in a total of 12 different methods used to measure the same underlying biological response (Fig. 1a). We repeated the entire process in biological triplicate (Supplementary Fig. 6).

Each of the 12 methods can be described by a unique sequence of measurement steps for sample preparation, signal detection, and measurand. We used several different sample preparations, including two different antibiotic treatments to halt translation prior to flow cytometry detection of fluorescent protein (kanamycin, Kn, and chloramphenicol, Cm), and two different techniques for labeling RNA via in situ hybridization (FISH and HCR) (Supplementary Fig. 2). After sample preparation, we split samples between two different signal detection methods: flow cytometry (abbreviated “flow” in Figs. 1–5), and microscopy. Finally, we chose three different measurands for the level of gene expression per cell: whole-cell fluorescence from protein, whole-cell fluorescence from labeled RNA, and estimated RNA count per cell (“RNA #” in Fig. 1a, Supplementary Fig. 5). We refer to methods according to their unique combination of sample preparation, signal detection, and measurand. For example, “HCR flow protein” refers to a method consisting of HCR (sample preparation), flow cytometry (signal detection), and protein (measurand).

For single-cell distributions measured by all 12 methods (Supplementary Fig. 6), we evaluated performance with regard to resolvability (Fig. 1b) and relative bias (Fig. 2). Furthermore, to attribute performance to differences in measurement steps, we made pairwise comparisons between methods that differ by one step only (Figs. 3–6). In this manner, we were able to identify performance differences between methods and attribute those differences to steps in the measurement process.

### Evaluation of resolvability

A useful metric for resolvability will provide quantitative information about the degree and direction of overlap between distributions that are measured at different levels of gene expression. To quantitatively evaluate the resolvability for a given method, we calculate the Area Under the receiver operating characteristic Curve (AUC) between single-cell distributions measured at different levels of IPTG (Fig. 1b). AUC values are unitless and range from zero to one, with 0.50 indicating no resolvability (cells grown with different amounts of IPTG have completely overlapping distributions), 1.00 indicating complete resolvability in the expected direction (cells grown with more IPTG have a greater signal, with no overlap between distributions), and zero indicating complete resolvability in the unexpected direction (cells grown with more IPTG have a lower signal, with no overlap). To evaluate resolvability over the entire range of induction, we use the seven AUC values calculated from pairs of adjacent IPTG concentrations to create AUC profiles (Fig. 1a, b). We also calculate an average AUC as a summary statistic to compare overall resolvability between methods (Supplementary Fig. 7).

The different measurement methods exhibit a wide range of performance with regard to resolvability (Fig. 1b and Supplementary Fig. 9). Average AUC’s range from ≈0.55 (HCR Flow RNA) to ≈0.75 (Cm Flow Protein) (ranked from lowest to highest in Supplementary Fig. 7). AUC profiles for all methods typically have a maximum near the middle of the applied range of IPTG concentrations where the biological response changes most rapidly, and for some methods, these maxima approach near-perfect resolvability (Fig. 1b). Superior resolvability can be achieved using entirely different combinations of measurement steps. For example, “FISH Microscopy RNA count” has an AUC profile maximum of ≈0.87 (Fig. 1b). Flow cytometry measurement of fluorescent protein expression levels following RNA-labeling by HCR (“HCR Flow Protein” in Fig. 1) has a similar AUC profile maximum of ≈0.90 (Fig. 1b). These methods differ in all three measurement steps, and yet both methods exhibit excellent resolvability, illustrating that there is no single step evaluated in this study that is required for high resolvability. Subsequent sections contain a more detailed examination of how resolvability differences between methods can be attributed to various measurement steps.

### Evaluation of relative bias

To evaluate whether relative bias between methods influences conclusions about cellular function, we fit the dose-response curves measured with the inducible plasmid to the Hill equation:

$$Signal = offset + A\times \frac{{{x}^{n}}}{{{K}_{1/2}^{n}+{x}^{n}}}$$

where *Signal* is gene expression level, *x* is the concentration of stimulus (in this case, the inducer IPTG), *A* is the amplitude of the response, *K*_1/2_ is the concentration corresponding to the half-maximal response, *n* is the effective cooperativity, and offset is the gene expression level in the absence of stimulus. These Hill parameters are increasingly used as design constraints for tuning the response of engineered biological systems^24,25^. So, to evaluate the impact of bias, we compared the parameter values obtained for each of the 12 different measurement methods (Fig. 2, Supplementary Figs. 14–17). Normalization of gene expression to a living reference using RPU has previously been shown to enable comparability of promoter-strength measurements^22^ and composability of genetic circuits^19^. We fit both the raw data and RPU-normalized data to the Hill equation (Supplementary Fig. 8–11), and compared the resulting parameters estimated from each method.

To determine whether bias between methods affects the Hill parameter estimates (Fig. 2, Supplementary Figs. 12–15), we applied a Friedman test to calculate *p*-values for the null hypothesis of no method-to-method bias for each parameter. The resulting *p*-values (Supplementary Fig. 16) indicate little or no relative bias between methods for *K*_1/2_, RPU-normalized amplitudes, or RPU-normalized offsets. Interestingly, however, the analysis indicates a potential method-to-method bias for the effective cooperativity, *n* (*p*-values 0.011 and 0.082 for fits to raw and RPU-normalized data, respectively) (Supplementary Fig. 16). The primary source of this bias is attributed to measurand (RNA or protein), as described in the next section.

To determine whether there was a significant replicate-to-replicate bias, we applied the Friedman test a second time, to calculate *p*-values for the null hypothesis of no replicate-to-replicate bias (Supplementary Figs. 12–15). The results indicated a significant replicate-to-replicate variability for *K*_1/2_ (*p*-values 1.9 × 10^−4^ and 2.6 × 10^−4^ for fits to raw and RPU-normalized data, respectively). There was also a potential replicate-to-replicate bias for raw amplitude (*p*-value: 0.017), however this effect was mitigated by normalization to RPU (*p*-value: 0.23). Taken together, these results suggest that the source of replicate-to-replicate variability shifted the dose-response curve along its *x*-axis without affecting offset, cooperativity, or normalized amplitude. One possible explanation is the variability of the concentration of active IPTG used in different replicates, for example, from variations in stock aliquots.

### Attribution of performance to measurand

To assess how choice of measurand can influence measurement performance, we compared pairs of methods that differed only in their measurands for gene expression: fluorescent protein (M_1_) or labeled RNA (M_2_) (Fig. 3a). Because these pairwise comparisons between methods share the same sample preparation and signal detection method, any observed differences in measurement performance can be attributed to measurand choice (RNA or protein). For each method, we measured both protein and RNA in the same set of cells. Consequently, these comparisons of measurement performance are not subject to bias that might arise from differential sampling of cells from the original culture.

We found that resolvability depends on measurand, and the nature of this dependence can be directly coupled to sample preparation. For samples prepared using FISH, RNA measurements offer superior resolvability than fluorescent protein measurements; this holds true across detection methods (microscopy and flow cytometry) (Fig. 3b). Conversely, for samples prepared using HCR, measurements of fluorescent protein measurements have better resolvability than RNA measurements; this also holds true across detection methods (Fig. 3b). Considering that resolvability of labeled RNA was generally comparable between both FISH and HCR, these results suggest that HCR performs better than FISH for studies that require concurrent resolvability of both RNA and protein^26–28^.

We found two potential examples of systematic bias in Hill parameters between RNA-based and protein-based measurements. Estimates for *n* (raw and RPU-normalized) are consistently higher when protein is the measurand as opposed to RNA, suggesting a potential systematic difference between the dose-response curves measured with the two different measurands (Fig. 2 and Supplementary Fig. 14). Most estimates for RPU-normalized *A* were higher for protein than RNA (Fig. 2 and Supplementary Fig. 12). Replicate-to-replicate reproducibility of Hill parameter estimates for *K*_1/2_ and *n* is generally worse for most RNA measurements compared to their corresponding protein measurements, and did not improve after normalizing to RPU (Fig. 2 and Supplementary Figs. 13–14). One possible explanation is that the process of labeling RNA introduces variability, whereas labeling is not required to detect fluorescent proteins.

### Attribution of performance to signal detection

To assess how single-cell measurement performance can be influenced by the signal-detection method, we compared pairs of methods that differed only in their signal detection: flow cytometry (D_1_) or microscopy (D_2_) (Fig. 4a). Because these pairwise comparisons between methods share the same sample preparation and choice of measurand, differences in performance can be attributed to signal detection.

Compared to flow cytometry, microscopy generally exhibits superior resolvability. This holds true across IPTG concentrations, sample preparations, and measurands (Fig. 4b). This could potentially be due to several advantages of microscopy over flow cytometry, such as the ability to unambiguously exclude non-cellular signals, optimized excitation and emission settings, and increased signal integration time. Replicate-to-replicate variability of resolvability was higher for microscopy than flow cytometry. One possible explanation for microscopy’s higher variability is lower throughput (microscopy typically measures 10^2^ cells–10^3^ cells, whereas flow cytometry typically measures 10^5^–10^6^ cells), so quantitative differences between microscopy distributions may be less reproducible.

Signal detection methods generally do not exhibit a relative bias for parameterization of dose-response (Fig. 2 and Supplementary Figs. 12–15). One potential exception is RPU-normalized amplitude: nine out of eleven estimates are higher for flow cytometry methods compared to their corresponding microscopy methods, indicating a potential relative bias between detection methods (Fig. 2 and Supplementary Fig. 12). Considering that no other Hill parameters show evidence of bias between detection methods, a difference in RPU-normalized amplitude between flow cytometry and microscopy could be due to biases in the measurements of the samples required for RPU-normalization. The process of RPU-normalization itself may not be a reliable way to reduce uncertainty^23^, and as such, Hill parameters that are not affected by RPU-normalization (*K*_1/2_ and *n*) are more robust descriptors of dose-response.

### Attribution of performance to sample preparation

To assess how single-cell measurement performance can be influenced by sample preparation, we compared pairs of methods that differ only in sample preparation (Fig. 5a). Because these methods share signal detection methods and choice of measurand, differences in performance can be attributed to sample preparation. We examined several sample preparations including RNA labeling strategy [FISH (P_1_) versus HCR (P_2_)], antibiotic treatment for cytometric detection of protein [kanamycin (P_3_) versus chloramphenicol (P_4_)], and fluorescent protein detection before versus after in situ hybridization (P_3_ versus P_1,_ P_3_ versus P_2,_ P_4_ versus P_1,_ P_4_ versus P_2,_ in Fig. 1a).

We found that resolvability depends on RNA labeling strategy, and the nature of this dependence is coupled to measurand. Because all cells shared the same buffers for fixation and permeabilization (Supplementary Note 2), any differences between FISH and HCR are likely due to the hybridization step. For protein, HCR-treated cells show superior resolvability to FISH-treated cells, across both detection methods (Fig. 5b). One possible explanation is that the respective hybridization buffers for FISH and HCR have different effects on preserving signal from fluorescent protein^29^. For labeled RNA, FISH shows slightly higher resolvability than HCR when detected using flow cytometry, however FISH shows comparable resolvability to HCR when detected using microscopy (Fig. 5b). Performance differences between FISH and HCR for resolving labeled RNA could depend on hybridization efficiency, as described below. Importantly, for this work, we chose a short amplification time to optimize HCR sample preparation for single-transcript detection. Although it is beyond the scope of this work, longer amplification time can improve detection of HCR-labeled RNA by flow cytometry^26^, and might also improve its resolvability.

Hill parameters estimated from RNA labeled by FISH or HCR indicate potential bias between methods (Fig. 2, Supplementary Figs. 12–15). For example, 12 out of 14 estimates of *K*_1/2_ are higher for HCR than FISH, suggesting potential relative bias between the two RNA labeling methods. In addition, parameters estimated from HCR-labeled RNA tended to have higher uncertainties, and, for some replicates, deviated substantially from all other methods. Parameters estimated from FISH-labeled RNA had lower uncertainties and tended to agree more with other methods, suggesting that FISH performed better than HCR for modeling dose-response from labeled RNA.

We also compared two different antibiotic treatments on live cells for flow cytometry measurements of fluorescent protein. We found that kanamycin (Kn, P_3_) and chloramphenicol (Cm, P_4_) generally exhibit good agreement in performance. Resolvability and Hill parameters were approximately the same, with slight differences possibly due to the timing of the measurements (Figs. 1b and 2, Supplementary Note 3, Supplementary Fig. 17).

To evaluate whether the hybridization process affects method performance, we compared cytometry measurements of fluorescent protein before versus after hybridization. Resolvability is lower following in situ hybridization by FISH or HCR, with a greater loss for FISH than HCR (Supplementary Fig. 18). Hill parameter estimates from cytometry measurements of protein were approximately the same before and after hybridization (Fig. 2, Supplementary Figs. 12–15). These results show that measurements of fluorescent protein following FISH or HCR are potentially useful for estimating Hill parameters, albeit at a lower resolvability of cellular response.

### Benchmarking a new method for single-transcript detection

FISH is an established approach for fluorescently labeling and counting individual RNA transcripts in cells^4,5,21^. New methods for fluorescently labeling individual RNA transcripts in cells continue to emerge^7,20,30^, however, these methods are rarely benchmarked against FISH. To demonstrate how single-transcript measurement performance can be benchmarked against FISH, we compared resolvability and bias between FISH and HCR for estimates of transcript counts per cell (Fig. 6a–g). HCR offers several potential advantages over FISH such as background suppression, amplifiable signal, and the ability to discern single-nucleotide variants^20,31^. Because HCR had not previously been used for transcript-counting in bacteria, we first optimized several aspects of the HCR sample preparation protocol, including starting cell culture volume, permeabilization agent, permeabilization temperature, permeabilization time, and amplification time (Supplementary Note 2). However, we note that we have not performed a comprehensive exploration of variables that could influence the performance of in situ hybridization for single-transcript detection^29^. Interestingly, converting from total RNA fluorescence per cell (M_2_) to estimates of RNA counts per cell (M_3_) subtly influences measurement performance. For example, for both FISH and HCR, converting from whole-cell fluorescence to estimated RNA counts per cell increases resolvability, and slightly changes Hill parameter estimates (Figs. 1 and 2, Supplementary Figs. 12–15 and 19).

Estimates of RNA count per cell directly indicate a bias between RNA-labeling methods, with FISH giving a twofold to threefold higher RNA count per cell than HCR (Supplementary Fig. 6). This bias in RNA count directly carries over to relative bias in the Hill equation amplitude and offset (Supplementary Figs. 8, 12, and 15). Furthermore, the relationship between expression level and noise in RNA counts is consistent with a relative difference in hybridization efficiency of 35.9% (HCR/FISH, Supplementary Fig. 20). In spite of this difference, FISH and HCR exhibit comparable resolvability (Fig. 6b), leading to the somewhat paradoxical observation that more efficient hybridization of target RNA transcripts with labeled probes does not necessarily equate to a superior ability to resolve changes in gene expression across the range of induction.

To further evaluate the effects of bias between FISH and HCR with regard to conclusions about cellular function, we compared estimates of transcriptional burst kinetics for FISH and HCR. Assuming a two-state model of transcription, we fit each distribution of RNA count per cell to a negative binomial, and estimated burst size and frequency as previously described^21^. The relationship between IPTG concentration and transcriptional burst parameters showed similar trends for FISH and HCR (Fig. 6e–g). However, burst frequency was generally lower for FISH than HCR (*p*-value: 6.6 × 10^−3^, Fig. 6e), and burst size was generally higher for FISH than HCR (*p*-value: 3.0 × 10^−6^, Fig. 6f). Because differences between FISH and HCR are mostly consistent with a difference in hybridization efficiency (Supplementary Fig. 20), we considered whether including an additional parameter for hybridization efficiency would improve agreement between the two methods. Hybridization efficiency impacts estimates of burst size but not burst frequency. Assuming 95.0 and 34.1% hybridization efficiencies for FISH^4^ and HCR (see “Methods”), respectively, including hybridization efficiency greatly improved agreement between FISH and HCR for estimating burst size (*p*-value: 0.15, Fig. 6g).

Our estimates of burst size and frequency are comparable to previous studies that use the same two-state promoter model to interpret single-molecule RNA FISH measurements of IPTG-induced transcription in *E. coli*^21,32^ (Supplementary Figs. 21 and 22). Differences between burst parameter estimates can possibly be attributed to differences between experimental conditions used in each study, which underscores the challenge of comparing estimates of biological function between different studies. This challenge of comparability highlights the tradeoffs and advantages of split-sample benchmarking as we have demonstrated here: although performing experiments with HCR and FISH in parallel came at a cost, in a single study we are able to directly compare transcriptional burst parameters between two different RNA labeling strategies performed under the same experimental conditions.

## Discussion

Using BRASS, we have shown how the performance of single-cell and single-transcript measurements can be compared while maximizing the extent to which experimental variability is shared between methods, and how performance differences can be systematically attributed to general steps throughout the measurement process. Furthermore, we have shown the utility of this approach for demonstrating new methods in parallel with traditional methods in order to benchmark method performance. We anticipate that the methods demonstrated here will directly enable measurements of fundamental importance to many biological systems. For example, cytometry-based detection of RNA remains an under-utilized tool for high-throughput, single-cell estimates of transcription dose-response, and we have demonstrated this capability for both FISH and HCR. Furthermore, the resolvability of cytometry-based detection of RNA suggests the feasibility of high-throughput measurements of RNA degradation rates in single cells without the need for RNA extraction, which remains a measurement need in a range of cell types^33^. Finally, our comparison of FISH to HCR highlights the importance of including hybridization efficiency in analysis of single-transcript measurements, which is a simple but critical step that is not always included in the analysis process.

As an alternative approach to split samples for comparing RNA labeling strategies in a single study, the same cells could be labeled by both FISH and HCR. This could potentially be done using sequential labeling of the same sample, utilizing DNAse treatment to strip one label before another label is used for analysis^34^. Or, direct comparison of FISH to HCR could be achieved using simultaneous labeling by designing probes that hybridize to different regions of the target mRNA^35–37^. However, these kinds of method comparisons assume that DNAse treatment or mRNA target sequence do not affect method performance, whereas a split-sample comparison does not require such assumptions.

While the focus of this work was to demonstrate a way to compare methods using a model system of bacterial sense and response, BRASS can potentially be used to compare methods for studying more complex systems. For example, single-cell microscopy has been used to analyze how well cells resolve stimulus and transmit information in eukaryotic signaling cascades^38^, however the influence of measurement methods on bias and resolvability is typically not addressed in such studies. BRASS could be used to compare experimental methods for analyzing such systems. In the most general sense, we anticipate that a better understanding of bias and resolvability in single-cell methods will directly improve our understanding of complex biological function.

Although this work was focused on evaluating fluorescence-based methods, BRASS is a generalizable framework to evaluate and compare any measurement methods in which a change in input stimulus changes a measured distribution of response. For example, single-cell RNA sequencing can be used to estimate transcription burst size and frequency^39^, however these measurements would benefit from a within-sample comparison to FISH to separate biological from measurement noise^1,40^. Other sequencing measurements, such as sort-seq^41^, rely heavily on resolvability, and BRASS could be used to understand the influence of resolvability on downstream applications. In another example, advances in single-molecule instrumentation have enabled simultaneous measurement of force and fluorescence distributions on the same sample^42^, and BRASS could be applied to compare resolvability and bias between these different measurands. Finally, this approach to attributing performance to measurement steps is compatible with the fractional-factorial design of experiments, which could be used to develop and evaluate methods in virtually any field.

## Methods

### Strains and plasmids

All experiments were performed with *Escherichia coli* strain NEB 10-beta (New England Biolabs, MA, C3019) containing one of three plasmids. Plasmid pAN1201 does not encode eYFP, and served as a negative control. Plasmid pAN1717 encodes and constitutively expresses eYFP, which served as a positive control as well as the benchmark for RPU. Plasmid pAN1818 encodes an inducible expression system that expresses eYFP from the P_tac_ promoter in the presence of isopropyl β-D-1-thiogalactopyranoside (IPTG, Supplementary Fig. 1).

### Probe design

FISH and HCR detection systems both used the same fluorophore: single-isomer 6-TAMRA. TAMRA-labeled FISH probes were designed using the Stellaris probe designer and are listed in Supplementary Table 3. HCR v3.0 probes and TAMRA-labeled hairpin amplifiers were designed with assistance from Molecular Technologies and are listed in Supplementary Table 3.

### Growth protocol

Glycerol stocks containing each of the 3 constructs (pAN1201, pAN1717, and pAN1818) were each streaked onto LB agar plates containing kanamycin (50 μg/mL) and grown overnight in a 37 °C incubator. Single colonies were used to inoculate 3 mL of M9 minimal media supplemented with 5% glucose, casamino acids (0.2%), Vitamin B1 (Thiamine, 0.34 g/L), and kanamycin (50 μg/mL) (referred to as “growth medium”) in a 14 mL culture tube and grown overnight for 16 h shaking and incubating at 37  °C. These overnight cultures were then diluted 1:300 into a final volume of 20 mL growth medium in a 50 mL Falcon tube. The overnight culture of pAN1818 was used to inoculate eight different cultures for each IPTG concentration (0, 5, 10, 20, 40, 100, 400, 1000 μmol/L). These ten total cultures (pAN1201, pAN1717, and pAN1818 at eight concentrations of IPTG) were grown for ~3.5 more hours, shaking and incubating at 37 °C until they reached an optical density at 600 nm (OD_600_) of ≈0.2 (actual values 0.22 ± 0.01 OD_600_, mean and standard deviation of all 10 samples across all three biological replicates), at which point they were placed on ice for subsequent sample preparation for flow cytometry or microscopy.

### Optimization of HCR protocol for single-transcript detection in bacteria

To develop a reproducible protocol for single-transcript detection in bacteria by HCR, we started with a recommended protocol for *E. coli* cells in suspension^20^. Many factors are known to affect in situ hybridization, and we tested several of these including fluorophore choice, starting culture volume, buffer composition, permeabilization conditions, hairpin preparation, and more. We used the following qualitative criteria for HCR protocol optimization: (1) a typical field of view should contain a sufficient number of cells for analysis (on the order of 10^1^ cells–10^3^ cells), (2) there should be minimal non-specific fluorescence and debris, and (3) the fluorophore should exhibit minimal bleaching over exposure times needed for signal detection. We found that at least one mL of culture is needed for enough cells to be analyzed at the end of the preparation. We found that permeabilization at 4 °C was preferable to 20 °C. We noticed no difference between snap cooling the hairpins on ice versus room temperature. Pipetting into new tubes after incubation steps minimized debris and non-specific fluorescence. Finally, with increased exposure time, AlexaFluor 546 and AlexaFluor 594 photobleached more than the single isoform 6-TAMRA. Collectively, these optimizations led to a protocol that yielded a sufficient number of cells to be analyzed on a clean background, with minimal photobleaching.

Protocol development for fixation and permeabilization was chosen to be compatible with split-sample labeling by either HCR or FISH to facilitate a direct comparison of the two techniques at the labeling step. Cells influenced by nonspecific extracellular fluorescence were systematically excluded in the analysis phase. Further optimization of these protocols might influence the results presented here^29^, and comprehensive exploration of protocol variables is the subject of ongoing work.

### Fixation and permeabilization of bacteria for labeling transcripts by in situ hybridization

From each culture, 6 mL was used for FISH and 6 mL was used for HCR. Samples were centrifuged for 10 min at room temperature at 4000 × *g*. After removing the supernatant, the cell pellet was resuspended in 750 μL of 1× PBS and transferred to a 1.5 mL microcentrifuge tube. To each tube, 250 μL of 4 % formaldehyde was added, and the samples were incubated for 16 hours overnight at 4 °C. Following incubation, the samples were centrifuged for 10 min at 4 °C. After removing supernatant, cell pellets were resuspended in 150  μl of 1× PBS and 850 μl methanol, and incubated at 4 °C for 3.5 h. Following this step, the cells were ready for transcript-labeling by either FISH or HCR.

### Transcript-labeling by FISH

After permeabilization, FISH labeling was performed as previously described^19,21^ with minimal exceptions as noted below. Briefly, cells were washed by centrifugation, removal of supernatant, and resuspension in Wash Buffer A (Biosearch Technologies, SMF-WA1-60) with 50% formamide. Cells were then washed and resuspended in 50 μL Hybridization Buffer (Biosearch Technologies, SMF-HB1-10) with 50% formamide containing probes at 62.5 μM, and left to incubate overnight at 30 °C. The next day, cells were washed three times with Wash Buffer A with incubations at 30 °C for 30 min in between each centrifugation. To label DNA, the third resuspension in Wash Buffer A contained diamidino-2-phenylindole (DAPI) at 10 μg/mL. Following incubation with DAPI at 30 °C for 30 min, cells were washed and resuspended in Wash Buffer B (Biosearch Technologies, SMF-WB1-20). Finally, cells were washed and resuspended in 50 μL 2× sodium chloride sodium citrate (SSC) and stored in the dark at 4 °C before imaging.

### Transcript-labeling by HCR

Following fixation and permeabilization as described above, 1 mL of cells were transferred into a new 1.5 mL Eppendorf tube and centrifuged for 5 min at 4 °C at 4000 × *g*. After removing the supernatant, cell pellets were washed by resuspension with 0.5 mL of 1× PBST buffer, following by centrifugation and removal of the supernatant. Probe hybridization buffer was pre-heated to 37 °C before use. Cells were re-suspended with 400 μL of probe hybridization buffer and incubated for 30 min at 37 °C. During this time, probe solution was prepared by adding 2 pmol of each probe mixture (“odd” and “even”, corresponding to the 5′ and 3′ halves of each target region on the RNA, 1 µL of 2 µmol/L stock per probe mixture) to 100 µL of probe hybridization buffer at 37 °C. Probe solution was added directly to each sample to reach a final probe concentration of 4 nmol/L. Samples were incubated overnight at 37 °C. The next day, probe wash buffer was pre-heated to 37 °C before use. 1 mL of probe wash buffer was added to each sample, and then centrifuged at room temperature at 4000 × *g* for 5 min. After removing supernatant, the cell pellet was resuspended with 500 µL wash buffer, incubated for 5 min at 37 °C, and centrifuged at room temperature at 4000 × *g* for 5 min. This step was repeated two more times, but with 10 min incubations in between centrifugation. The cells were then ready for amplification.

Amplification buffer was equilibrated to room temperature before use. Cells were resuspended with 150 µL amplification buffer and incubated for 30 min at room temperature. TAMRA-labeled hairpins were prepared by heating 5 µL of 3  µmol/L stock to 95 °C for 90 s, and allowing them to cool to room temperature in the dark for 30 min. Hairpin mixture was prepared by adding all of the cooled hairpins to 100 µL of amplification buffer at room temperature. The hairpin mixture was added directly to each sample to reach a final hairpin concentration of 60 nmol/L. Samples were incubated for 45 min in the dark at room temperature, before adding 1 mL of 5× sodium chloride sodium citrate with 0.1% Tween 20 (SSCT) buffer. Samples were centrifuged at room temperature at 4000 × *g* for 5 min, and then the supernatant containing the hairpin solution was removed. Cells were resuspended with 500 µL of 5× SSCT with DAPI (1 µL of 5 µg/mL stock) and incubated for 5 min at room temperature. Samples were then centrifuged at room temperature at 4000 × *g* for 5 min, and the DAPI solution was removed. The cell pellet was resuspended in 5× SSCT buffer, incubated for 5 min at room temperature, and then the supernatant was removed. This wash step was repeated two more times except with 10 min incubations in between centrifugation. Finally, the cell pellets were resuspended in 50 µL of 5× SSC buffer, and the samples were stored in the dark at 4 °C before imaging.

### Flow cytometry before and after transcript-labeling

Flow cytometry measurements were made with *E. coli* samples both before and after fixation and labeling. For samples measured before fixation, cells grown to mid-log phase (OD_600_ of 0.2, see Methods) were diluted 1000-fold into 1× PBS buffer containing either kanamycin (2 mg/mL) or chloramphenicol (170 μg/mL) to halt translation. For samples measured after fixation and labeling, samples were diluted after the final step of the FISH or HCR labeling protocol 5000-fold into 5× SSC buffer for initial flow cytometry. For all samples an initial flow cytometry measurement was made to determine sample cell concentration and samples were diluted in accordance with this measurement to achieve ~10^5^ cell counts (events) per 150 μL sample draw volume for analysis. Measurements were made between 60 and 120 min after treatment with antibiotics for samples before fixation. FISH and HCR samples were measured the same day the labeling process was completed.

Flow cytometry measurements were made using an Attune NxT cytometer equipped with a 96-well plate autosampler. The detector gains for forward scattering and side scattering were both set at 350 V. The detection threshold was set to 200 for the forward scattering channel and 300 for the side scattering channel; these threshold levels were chosen to minimize the number of background (non-cell) events while ensuring that nearly all of the cells were detected. Cytometry data were collected for a sample volume of 150 μL for each sample, with a flow rate of 100 μL/min. The resulting number of singlet cell events for each sample ranged from 127,242 to 245,090 for cells measured before fixation, and from 34,238 to 140,014 for cells measured after fixation and labeling. For each sample, both the eYFP and TAMRA signals were measured. The eYFP signal was measured with 488 nm excitation laser and a 530 nm ± 15 nm bandpass emission filter. TAMRA signal was measured with 561 nm excitation laser and a 585 nm ± 8 nm bandpass emission filter. Blank samples were measured with each set of *E. coli* samples, and the results of the blank measurements were used with an automated gating algorithm to discriminate cell events from non-cell events (Supplementary Fig. 3). A second automated gating algorithm was used to select singlet cell events and exclude doublet, triplet, and higher-order multiplet cell events (Supplementary Fig. 3). All subsequent analysis was performed using the singlet cell event data.

For HCR Flow RNA, replicate 3, two samples (pAN1818 with 40 μmol/L IPTG, and pAN1717) were excluded due to a flow cytometer malfunction.

### Microscopy

Following FISH or HCR labeling, samples were imaged as described previously^19,21^ with minimal exceptions as noted below. Briefly, 2 µL of the sample was pipetted onto a #1 borosilicate glass coverslip (45 mm × 50 mm, Fisher Scientific, #12-544 F). A 1.5% agarose gel pad was placed on top of the sample droplet to keep the cells close to the imaging surface, and another #1 borosilicate glass coverslip (22 mm × 22 mm, Fisher Scientific, #12-545B) was placed on top of the agarose pad. Samples were imaged with an inverted epifluorescence microscope (Zeiss Axio Observer.Z1) using a 100 × 1.46 N.A. oil immersion phase contrast objective lens (Zeiss, alpha Plan-Apochromat Ph3 M27) and a sCMOS camera (Hamamatsu Orca Flash 4.0). Hardware control and image acquisition used Zen Pro Software (Zeiss). For each image, channels were imaged from longest to shortest excitation wavelength to minimize photobleaching from cross-talk between imaging channels. TAMRA-labeled RNA was imaged using an HXP 120 W mercury arc lamp at 100% intensity for excitation, with a 550 ± 12 nm excitation filter, a 570 nm beamsplitter, a 605 ± 35 nm emission filter, and 1 s integration time for each of 9 z-slices separated vertically by 200 nm (total z-range 1.6 μm). eYFP was imaged using a 470 nm LED light source set to 100% (Zeiss, Colibri), a 470 ± 20 nm excitation filter, a 495 nm beamsplitter, a 525 ± 25 nm emission filter, and an integration time of 1000 ms for 1 z-slice. DAPI-stained DNA was imaged using 385 nm LED excitation at 25% intensity (Zeiss, Colibri), a 359 ± 24 nm excitation filter, a 395 beamsplitter, and a 445 ± 25 nm emission filter with an integration time of 50 ms. Lastly, the bacterial cell bodies were imaged by phase contrast using a transmitted light halogen lamp set to 4 V, with an integration time of 100 ms for each of 9 z-slices separated by 200 nm, for a total z-range 1.6 μm. Each slide preparation was imaged at multiple locations across the agarose pad. If needed, multiple slide preparations were used to collect a total of at least 300 cells per sample.

### Microscopy image analysis

Image processing was performed as previously described^19,21^ with minimal exceptions except as noted. Using ZEN Pro software (Zeiss), microscopy image files were exported as TIFFs without compression. Images shown in the manuscript all using the following settings for each channel: Phase contrast, Black 2500 – White 7500; DAPI, Black 100 – White 200; TAMRA: Black 750 – White 3000; eYFP: Black 300 – White 4000. To minimize effects that could arise from uneven illumination of the sample at the edges of a field of view, cells were only analyzed within a rectangular region of interest in the center of each image. Phase-contrast images were used for cell segmentation, as previously described using Schnitzcells^43^. Spot detection was performed as previously described using Spatzcells^21^. This program quantifies the location and fluorescence intensity of diffraction-limited spots in the following manner. Gaussian smoothing is applied to reduce the contribution of pixel-to-pixel noise. 2D local maxima are detected in each z-slice, and then maxima are matched between z-slices. The locations of these maxima are fit to 2D Gaussian functions.

Due to subtle variations and occasional drift in z-focus during image collection, not all images were collected at the same focal plane, resulting in a small subset of frames that were not focused on the middle plane of the bacteria. To eliminate this source of variability from downstream analysis, distributions of TAMRA signal/cell as a function of z-slice were used to manually identify and exclude out-of-focus images that did not have a peak intensity in the middle third of the z-stack (slices 4–6). Due to cell-to-cell variability in fixation and permeabilization efficiencies, some cells are better prepared for in situ hybridization than others. To measure and account for this source of variability, DAPI was used to non-specifically stain DNA within each cell. Cells with a DAPI signal less than half the mean for that sample were excluded. In this manner, only those cells whose DNA was readily available for staining were included in subsequent analysis of RNA and protein fluorescence. Artifact exclusion for microscopy was necessary because non-specific fluorescence outside of cell bodies were occasionally in close enough proximity to the cells to affect the fluorescence within the segmented cell. Cells were visually inspected to exclude the influence of non-specific fluorescence on image analysis. Some cells were located near non-specific fluorescence that originated outside of the cell body. These cells were excluded from the analysis. Cells for which fluorescence originated from spots inside of the cell body were included in the analysis.

Because the RNA transcript was relatively short (720 nt), there was overlap in spot brightness distributions for non-specific and specific signal. A mixture of 2 lognormal distributions constrained to be centered on either side of a threshold intensity was used to fit the spot intensity histograms, to estimate the total spot intensity due to specific signal. Because spots cannot always be spatially separated from one another in diffraction-limited microscopy, we use a calibration method to estimate the number of spots per cell area based on the integrated brightness of spots per cell area, after correcting for background subtraction. This method is described in detail in ref. ^19^. In this manner, we are able to use integrated spot brightness to account for spots that cannot be spatially separated in diffraction-limited microscopy.

### Normalization to RPU

Converting expression levels to RPU by normalization to constitutive expression from the J23101 promoter enables comparability of Hill amplitudes in the same units, and has been shown to reduce variability in promoter strength measurements across different growth conditions^19,22,44^.

Normalization to RPU was performed in the following manner, where “Signal” indicates the median of a measured distribution of the indicated sample:

$${\mathrm{RPU}}=\,\frac{{\mathrm{Signal}}_{{\mathrm{pAN1818}}}-\,{\mathrm{Signal}}_{{\mathrm{pAN1201}}}}{{\mathrm{Signal}}_{{\mathrm{pAN1717}}}-\,{\mathrm{Signal}}_{{\mathrm{pAN1201}}}}$$

Here, pAN1818 is a sample used to measure inducible gene expression, pAN1717 is a sample used to measure constitutive gene expression from the J23101 promoter, and pAN1201 is a sample lacking the expression system (used as a negative control to measure non-specific signal).

### Hill equation parameterization

Response to extracellular concentrations of IPTG (*x*) for a given measurement output (Signal) were fit to a Hill equation. For every measurement method from each of the three replicates, we calculated mean, median, and geometric mean as estimates of distribution location parameters, and we estimated uncertainty using bootstrapping of the raw distributions. We found little difference between mean, geometric mean, and median; median was chosen to reduce effects from outliers or distribution tails. Fits were performed using the medians of the distributions with bootstrapped uncertainty using 1000 iterations. Residuals from the Hill fits did not show systematic error, indicated that the Hill equation sufficiently captured the dose-response trends for all methods, but bootstrapping generally underestimates variability around the Hill fit. The inverse variance was constrained to lie within a factor of 10 below or above the geometric mean of the available inverse bootstrapped variances within a replicate of the pAN1818 samples, and was used to weight a nonlinear least-squares fit. In all cases, the stated uncertainty on Hill parameters represents 95% confidence intervals of this fit.

### Friedman test for assessing method-to-method and replicate-to-replicate effects

The Friedman test is a non-parametric test to assess whether the ordering of parameter values across a primary factor (e.g., methods) is more consistent across multiple instances of a blocking factor (e.g., biological replicates) than would be expected to occur under random permutations within each instance of the blocking factor. For each of the Hill parameters, we assessed potential biases among methods by applying the Friedman test with the measurement method as the primary factor and biological replicate as the blocking factor. For each of the Hill parameters, we also assessed potential biases among biological replicates by applying the Friedman test with replicate as the primary factor and measurement method as the blocking factor.

### Effect of different hybridization efficiencies on mean and Fano

Let *n*_*irsm*_ denote how many RNA molecules are in cell *i* in replicate *r* of sample *s* to be measured by method *m*, and let *Y*_*irsm*_ denote the corresponding measurement. Suppose that the distribution of the number of RNA molecules per cell is fixed within sample type (i.e., IPTG concentration), such that $E({n}_{irsm})={\mu }_{s}$ and $Var({n}_{irsm})={\sigma }_{s}^{2}$. Suppose further that for method *m* each RNA molecule in any cell has an equal chance, *p*_*m*_, of being detected by hybridization, and that detections are independent of one another, such that ${Y}_{irsm}|{n}_{irsm} \sim {\mathrm{Binomial}}({n}_{irsm},{p}_{m})$. Then the following relationships hold:

$$E({Y}_{irsm})={\mu }_{s}{p}_{m}$$

$$Var({Y}_{irsm})={\mu }_{s}{p}_{m}(1-{p}_{m})+{p}_{m}^{2}{\sigma }_{s}^{2}$$

$${\mathrm{Fano}} ({Y}_{irsm}) = 1+{p}_{m}\left(\frac{{\sigma }_{s}^{2}}{{\mu }_{s}}-1\right)$$

Interestingly, these results imply that if two methods, say *A* and *B*, differ only by hybridization efficiencies, then

$$\frac{E({Y}_{irsA})}{E({Y}_{i^{\prime}rsB})}=\frac{{\mathrm{Fano}}({Y}_{irsA})-1}{{\mathrm{Fano}}({Y}_{i^{\prime}rsB})-1}=\frac{{p}_{A}}{{p}_{B}}$$

In Supplementary Fig. 20, we plot the log ratio of mean RNA counts per cell between HCR and FISH, and the log ratio of (Fano factor−1) between HCR and FISH, for each IPTG concentration, in each replicate. The 95% confidence intervals for each ratio were evaluated using 1000 bootstrapping iterations. The estimated ratios appear generally stable across biological replicate, IPTG concentration, and sample statistic type (i.e., Fano−1 or mean RNA count), supporting the hypothesis that differences in RNA counts from HCR and FISH can be primarily attributed to differences in hybridization efficiency. We then took the overall median ratio, across both Fano factor and mean, using all samples and all replicates, which produced an estimated hybridization efficiency ratio of 0.359:1 for HCR:FISH (Supplementary Fig. 20). For samples with no IPTG, replicates 1 (HCR) and 3 (FISH) had Fano factors <1, so they could not be included in this calculation which requires (Fano− 1) > 0 for both FISH and HCR. However, for those samples with no IPTG, ratios of their means were included in this analysis. In Fig. 6f, we assume 100 % hybridization efficiency for both FISH and HCR. In Fig. 6g, we assume a 95% hybridization efficiency for FISH as previously reported^4^, and we use the analysis above to calculate a hybridization efficiency for HCR relative to FISH (calculated HCR hybridization efficiency = 95% × 0.359 ≈ 34.1%).

### Parameterization of transcriptional bursting using negative binomial distributions

Previous studies have fit mRNA distributions with a variety of distribution models including negative binomials^32^, Poisson with a zero-burst mode for cells with no RNA^45^, or Poisson following exclusion of low-count cells^46^. We cannot justifiably exclude the contribution of cells with no RNA, since these cells pass our quality control check for “stainability” with DAPI (Supplementary Note 2), and they may result from a real biological contribution which must be accounted for in the analysis. For this reason, we chose an unmodified negative binomial to estimate transcription burst size and frequency as follows:

$$P(n)= \left(\begin{array}{c}xn+r-1\\ n\end{array}\right){p}^{r}{(1-p)}^{n}$$

where *r* and *p* are fitting parameters that can be used to estimate burst frequency (*f* = *r*/τ_RNA_, where τ_RNA_ is RNA lifetime) and burst size (*b* = (1−*p*)/*p*). In Fig. 6, the RNA lifetime is taken to be a constant equal to 2.8 min^47^. This treatment assumes the two-state model of transcription^48^ in which the promoter switches between an on state and an off state, and it produces multiple transcripts during the on state.

To assess the role of non-specific signals in our modeling of transcriptional bursting, we applied our model of transcriptional bursting to our negative-control samples. For both FISH and HCR, estimates of burst size and frequency from negative controls were similar to those from the lowest expression samples (0 IPTG) (Supplementary Figs. 21 and 22).

### Statistics and reproducibility

The names of all statistical tests and *p*-values are noted in the text. In general, we provide two-sided *p*-values for sign tests, except for *p*-values for Friedman tests, which are inherently one-sided. We do not rely on alpha values for drawing conclusions. The number of cells in every measurement is provided in Supplementary Table 4. The median was used for analyses that required estimating the central tendency of single-cell distributions. Uncertainty on the median was estimated using bootstrapping with 1000 iterations. Uncertainty on Hill parameters was estimated using 95% confidence intervals of nonlinear least-squares fit.

A total of three biological replicates were performed on different days. To clearly present replicate-to-replicate variability, results from each replicate are plotted separately in every figure.

As a general approach to processing, the inferences in this work treat the observations that remain after processing as a random sample of the population for characterization, which in this case is the entire population of *E. coli* cells growing in culture. We assume the filtering that has been done eliminates experimental artifacts and does not suppress or accentuate subsets of the general *E. coli* population. We do not incorporate any uncertainty due to potential biases, which if present, could dramatically increase uncertainty characterizations.

### Reporting summary

Further information on research design is available in the Nature Research Reporting Summary linked to this article.

## Supplementary information

Peer Review File

Supplementary Information

Description of Additional Supplementary Files

Supplementary Data 1

Reporting Summary

## Data Availability

Source data for figures are available through the NIST Data Science Portal (10.18434/mds2-2300)^49^ and Supplementary Data 1.
