# Supplementary material for: Comparison of bias and resolvability in single-cell and single-transcript methods
Source: Commun Biol. 2021 Jun 2;4:659. doi: 10.1038/s42003-021-02138-6 (PMC8172639; doi:10.1038/s42003-021-02138-6)
Supplement: Supplementary file 2 — Supplementary Information [file 42003_2021_2138_MOESM2_ESM.pdf]

# Comparison of bias and resolvability in single-cell and single-transcript methods

Jayan Rammohan*, Steven P. Lund*, Nina Alperovich*, Vanya Paralanov*, Elizabeth A. Strychalski*, David Ross*
*National Institute of Standards and Technology, 100 Bureau Drive, Gaithersburg, MD

## Supplementary Information

### Table of Contents

***Supplementary Figures*** ........ *3*

**Supplementary Figure 1. Fluorescent protein expression in bacteria was used as a model system for cellular response** ........ 3

**Supplementary Figure 2. FISH or HCR were used to label RNA** ........ 4

**Supplementary Figure 3. Flow cytometry gating example.** ........ 5

**Supplementary Figure 4. Multiple microscopy channels were used to simultaneously image cell bodies, DNA, RNA and protein.** ........ 6

**Supplementary Figure 5. Single-cell microscopy of cells containing RNA transcripts labeled either by FISH or HCR were used to estimate RNA counts per cell** ........ 7

**Supplementary Figure 6. A total of 12 single-cell measurement methods were used to measure distributions of cellular response across a range of stimulus, in triplicate.** ........ 8

**Supplementary Figure 7. Average AUC can be used to rank overall resolvability between methods.** ........ 9

**Supplementary Figure 8. Cellular response was quantitatively parameterized using Hill functions fit to raw medians of distributions** ........ 10

**Supplementary Figure 9. Residual error from Hill fits to raw medians** ........ 11

**Supplementary Figure 10. Cellular response was quantitatively parameterized using Hill functions fit to RPU- normalized medians** ........ 12

**Supplementary Figure 11. Residual error from Hill fits to RPU-normalized medians** ........ 13

**Supplementary Figure 12. Hill parameters for amplitude of raw and RPU-normalized response functions** ........ 14

**Supplementary Figure 13. Hill parameters for half-maximal induction for raw and RPU-normalized response functions** ........ 15

**Supplementary Figure 14. Hill parameters for effective cooperativity from raw and RPU-normalized response functions** ........ 16

**Supplementary Figure 15. Hill parameters for offset from raw and RPU-normalized response functions** ........ 17

**Supplementary Figure 16. Friedman test for relative bias between methods.** ........ 18

Supplementary Figure 17. Effect of antibiotic treatment on flow cytometry measurement of fluorescent protein prior to *in situ* hybridization. .... 19

Supplementary Figure 18. Comparison of flow cytometry detection of fluorescent protein before versus after *in situ* hybridization. .... 20

Supplementary Figure 19. Effect of calibrating whole-cell RNA fluorescence intensity to estimate RNA counts. .... 21

Supplementary Figure 20. Bias between FISH and HCR is consistent with a relative difference in hybridization efficiency .... 22

Supplementary Figure 21. Estimates of burst frequency. .... 23

Supplementary Figure 22. Estimates of burst size. .... 24

*Supplementary Tables* .... *25*

Supplementary Table 1. Growth protocol .... 25

Supplementary Table 2. DNA sequences used in this work. .... 26

Supplementary Table 3: FISH and HCR Probes used in this work .... 27

Supplementary Table 4: Number of cells for each measurement .... 28

*Supplementary Notes* .... *30*

Supplementary Note 1: General protocol for Bias and Resolvability Attribution using Split Samples (BRASS) .... 30

Supplementary Note 2: HCR protocol for single-transcript detection in bacteria .... 33

Supplementary Note 3: Attribution of performance to sample preparation (antibiotic treatment) . 35

Supplementary References .... 36

## Supplementary Figures

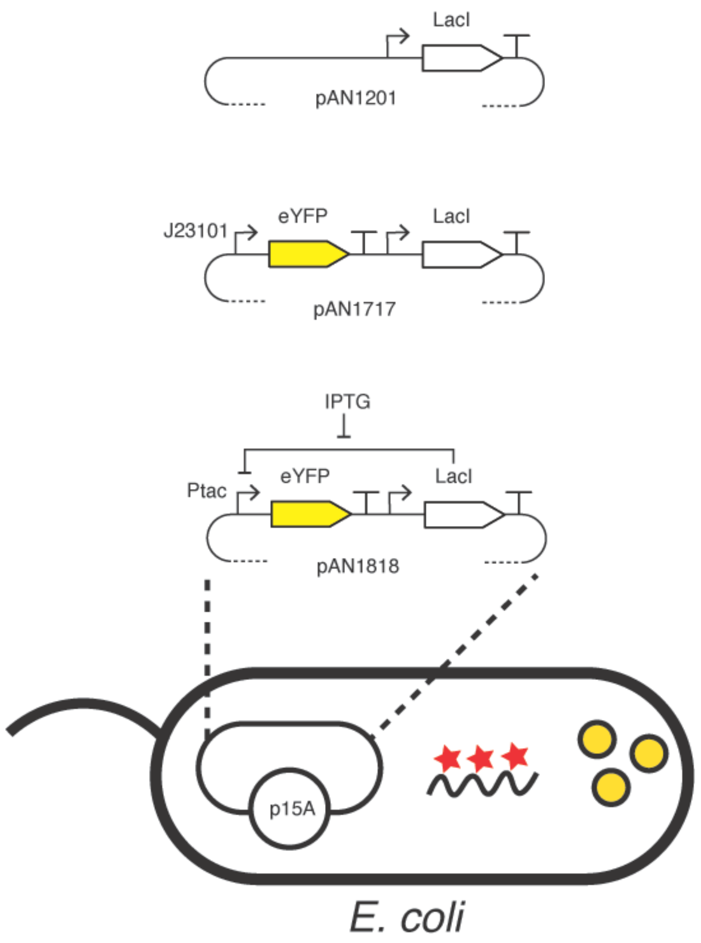


Supplementary Figure 1. Fluorescent protein expression in bacteria was used as a model system for cellular response. *Escherichia coli* (*E. coli*)was transformed with DNA plasmids from which RNA and fluorescent protein (yellow circles) were expressed. RNA was labeled with fluorophores using *in situ* hybridization (represented by red stars). A plasmid lacking the expression cassette was used as a negative control (top, pAN1201). A plasmid expressing eYFP from the J23101 promoter was used as a positivecontrol (pAN1717). A plasmid containing the $P_{tac}$ promoter was used for measuring induction of eYFP by IPTG (pAN1818).

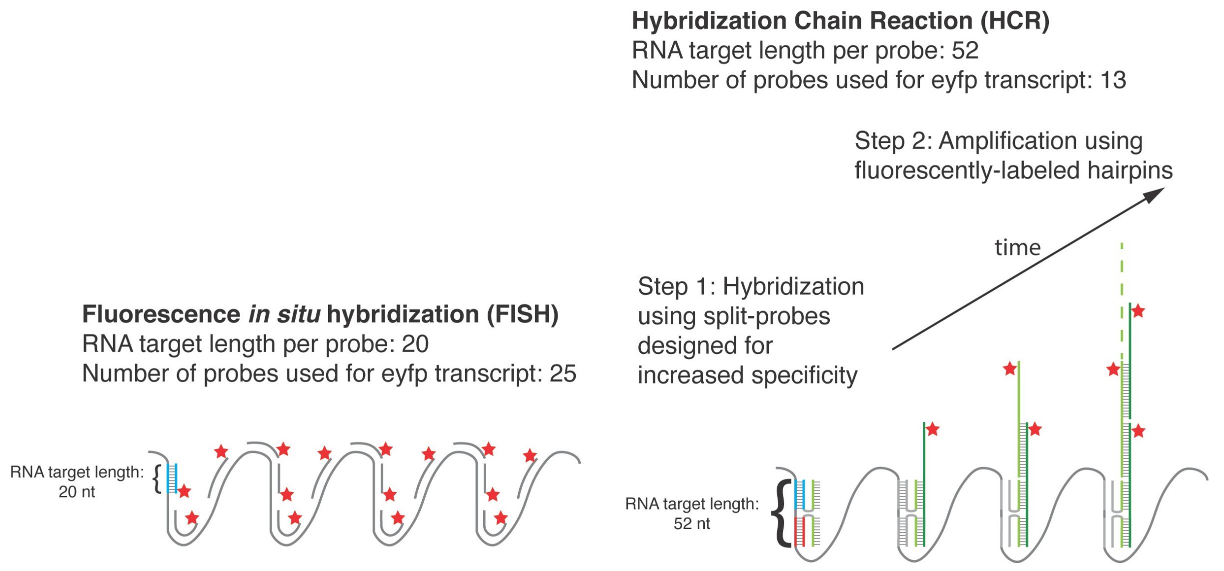


Supplementary Figure 2. FISH or HCR were used to label RNA. (Left) Traditional fluorescence *in situ* hybridization (FISH) uses multiple singly- labeled oligonucleotide probes hybridized along the length of a target RNA. In this study, we used 25 probes, each 20-nt in length and labeled with a single TAMRA fluorophore, targeted to the *eyfp* transcript. (Right) Hybridization chain reaction (HCR) (as described in Reference 6) uses a 2-step approach for labeling RNA. In Step 1, a split-probe is used to target the RNAand serve as an initiator site. In Step 2, TAMRA-labeled hairpins bind the initiator probe and amplify as part of a chain reaction over time. In this study, we used 13 split-probes to target the *eyfp* transcript. Each pair of split-probes targeted a 52-nt region of RNA.

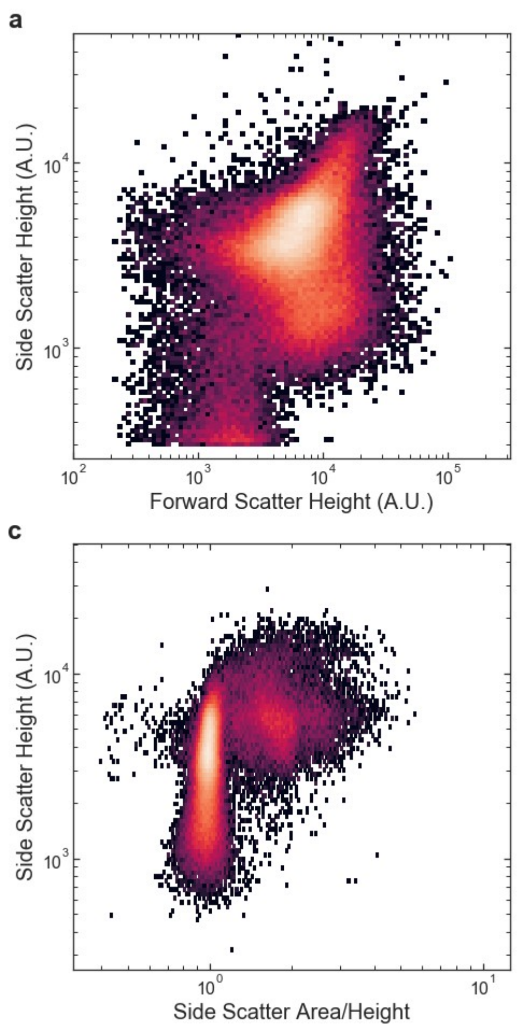


Supplementary Figure 3. Flow cytometry gating example. Flow cytometry gating example. **a**, Side scatter vs. forward scatter plot before automated cell gating, showing both cell and non-cell detection events. **b**, Side scatter vs. forward scatter plot after automated cell gating, showing only events most likely to be cell events. **c**, Side scatter vs. side scatter area/height plot before automated singlet gating, showing both singlet and multiplet cell detection events. **d**, Side scatter vs. side scatterarea/height plot after automated singlet gating, showing only singlet cell detection events.

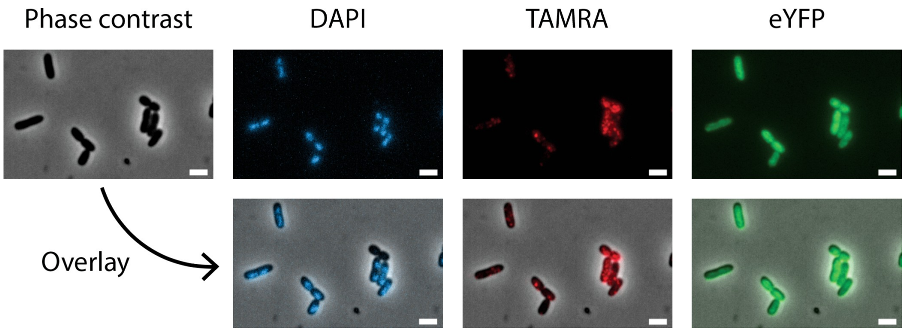


Supplementary Figure 4. Multiple microscopy channels were used to simultaneously image cell bodies, DNA, RNA and protein. Top row, left toright: Phase contrast was used for imaging cell bodies, DAPI was used to detect DNA, TAMRA-conjugated probes were used to detect FISH- or HCR-labeled RNA, and eYFP was used to detect protein expression. Bottom row: Phase contrast is shown overlaid with DAPI, TAMRA, or eYFP. Images shown are for pAN1818 grown in the presence of 100 μmol/L IPTG. Sample shown is pAN1818 grown in 100 μmol/L IPTG. Scale bar in the bottom right of each image is 2 μm.

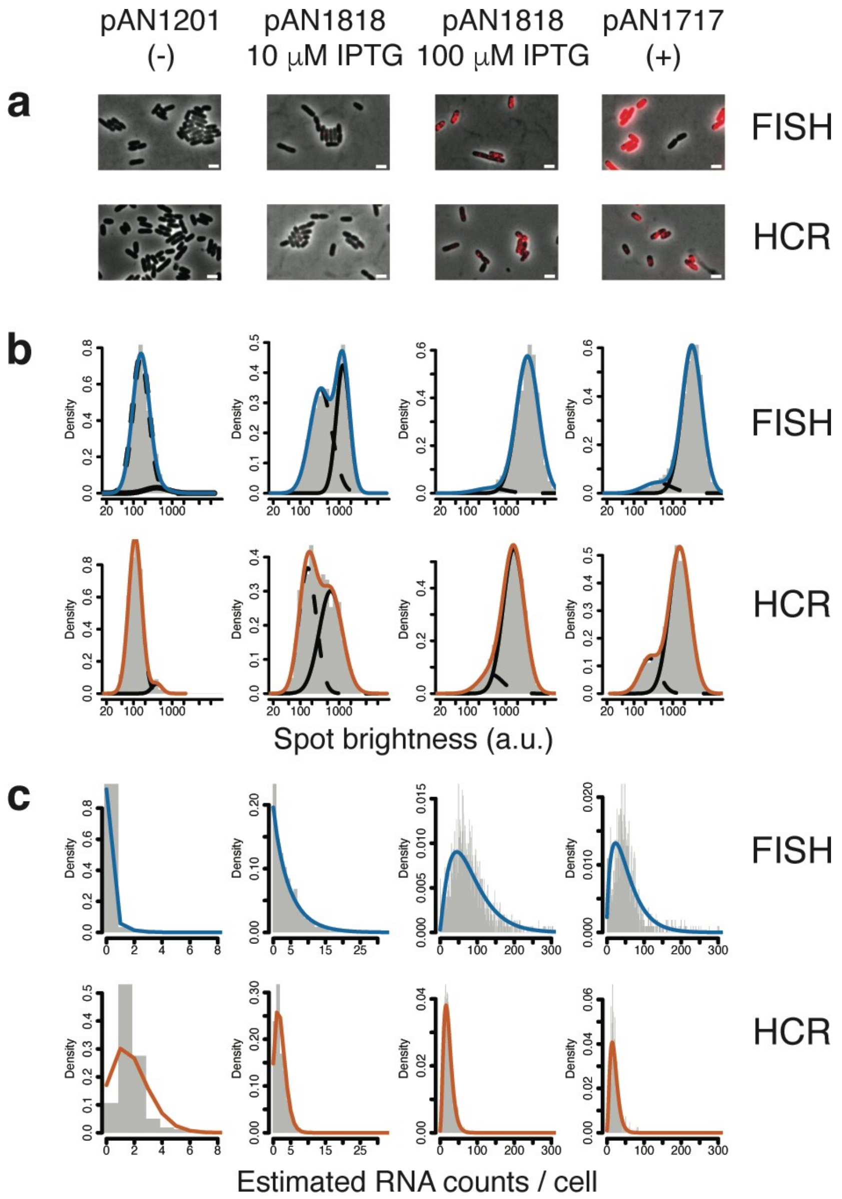


Supplementary Figure 5. Single-cell microscopy of cells containing RNA transcripts labeled either by FISH or HCR were used to estimate RNA counts per cell. Left to right for all panels: Negative control (pAN1201, no expression), low induced expression (pAN1818 with 10 μmol/L IPTG), higher induced expression (pAN1818 with 100 μmol/L IPTG) and positive control (pAN1717, constitutive expression from J23101 promoter). (a) Microscopy images with 2 μm scale bar. Top row FISH, bottom row HCR.

(b) Spot intensity histograms (gray bars), fit with two log-normal distributions (lower intensity, dashed black line; higher intensity, solid black line). Top row: FISH, Bottom row: HCR. (c) Estimated RNA counts per cell, fit with negative binomial distributions. Top row: FISH, Bottom row: HCR.

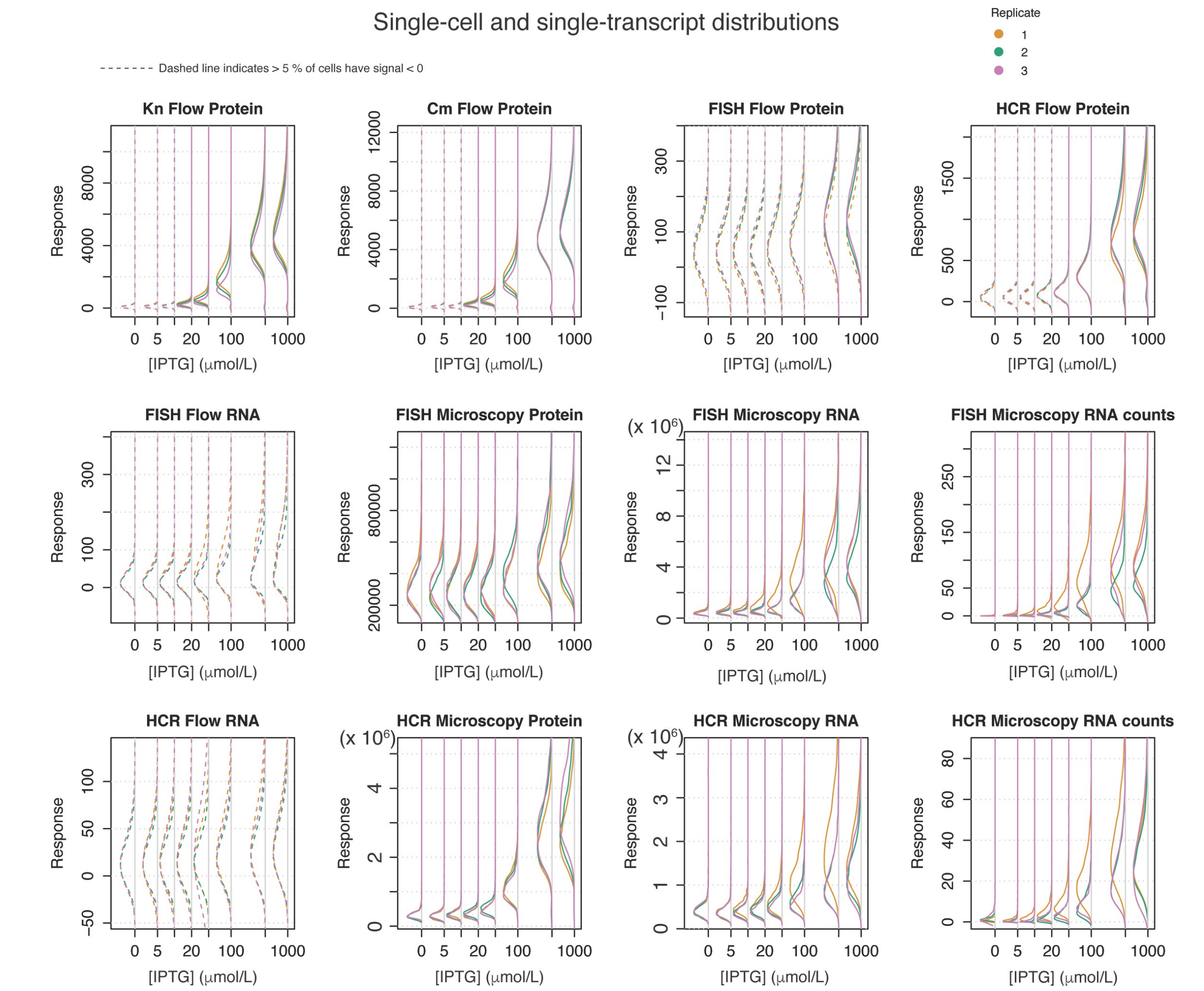


Supplementary Figure 6. A total of 12 single-cell measurement methods were used to measure distributions of cellular response across a range of stimulus, in triplicate. For all distributions, colors are used to distinguish biological replicate 1 (orange), biological replicate 2 (green), and biological replicate 3 (purple). Methods varied according to sample preparation (before fixation, treated with Kn or Cm; after fixation, RNA labeled with FISH or HCR), signal detection (microscopy or flow cytometry), andmeasurand (RNA or protein; for RNA, whole-cell TAMRA fluorescence was analyzed in addition to estimated RNA counts).Dashed lines indicate distributions for which > 5 % of the data lies below 0, after correcting for background signal in flow cytometry.

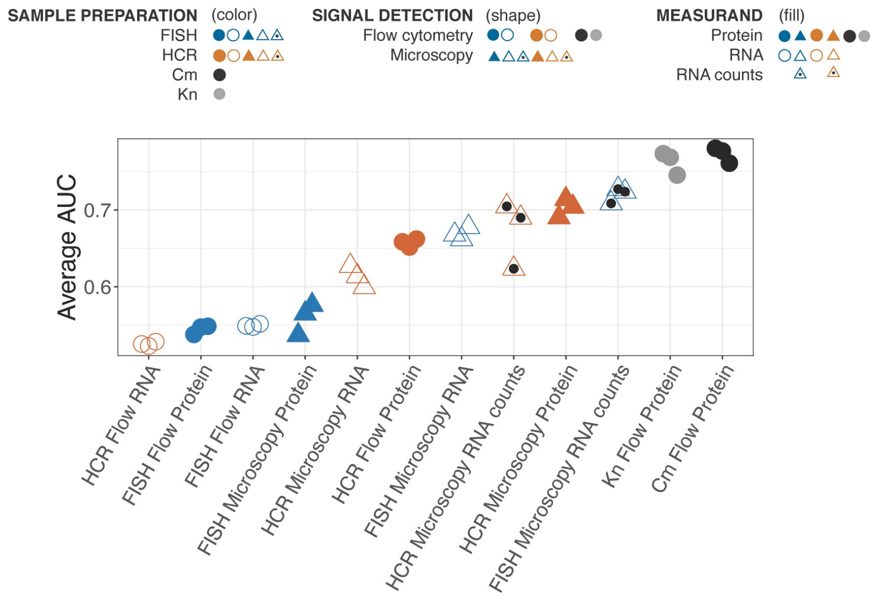


Supplementary Figure 7. Average AUC can be used to rank overall resolvability between methods. For each method, an average AUC was calculated from all seven values of the AUC profile. This was performed for each replicate. Methods are listed from left toright in order of lowest to highest average AUC.

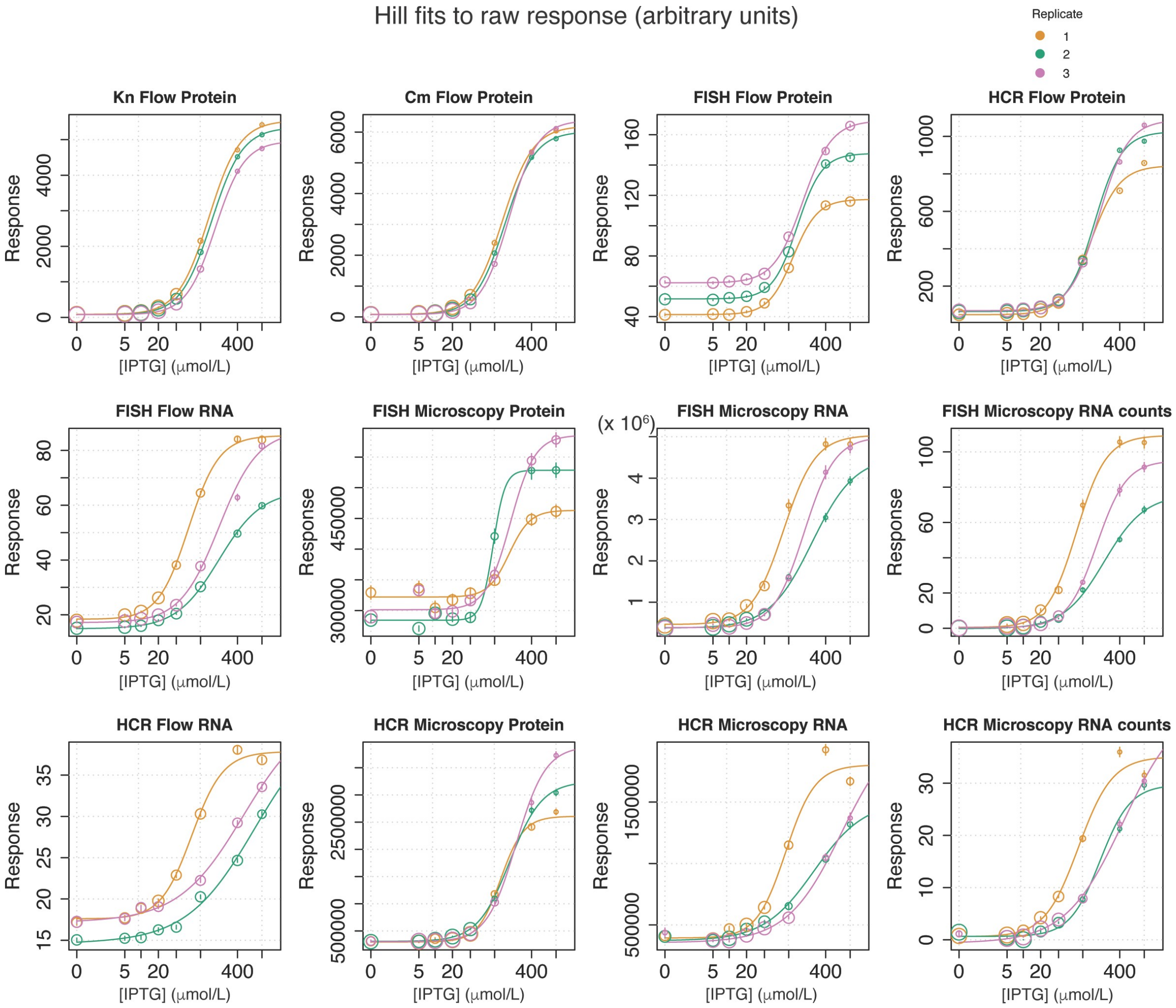


Supplementary Figure 8. Cellular response was quantitatively parameterized using Hill functions fit to raw medians of distributions. For all 12measurement methods, the raw medians (circles) were fit to a Hill function (lines) using weighted nonlinear least squares. The size of each circle is proportional to its weight in the fit, determined by its inverse variance. Error bars indicate 95 % confidence intervals of each median as determined by bootstrapping using 1,000 iterations. Color indicates biological replicate 1 (orange), replicate 2 (green), and replicate 3 (purple).

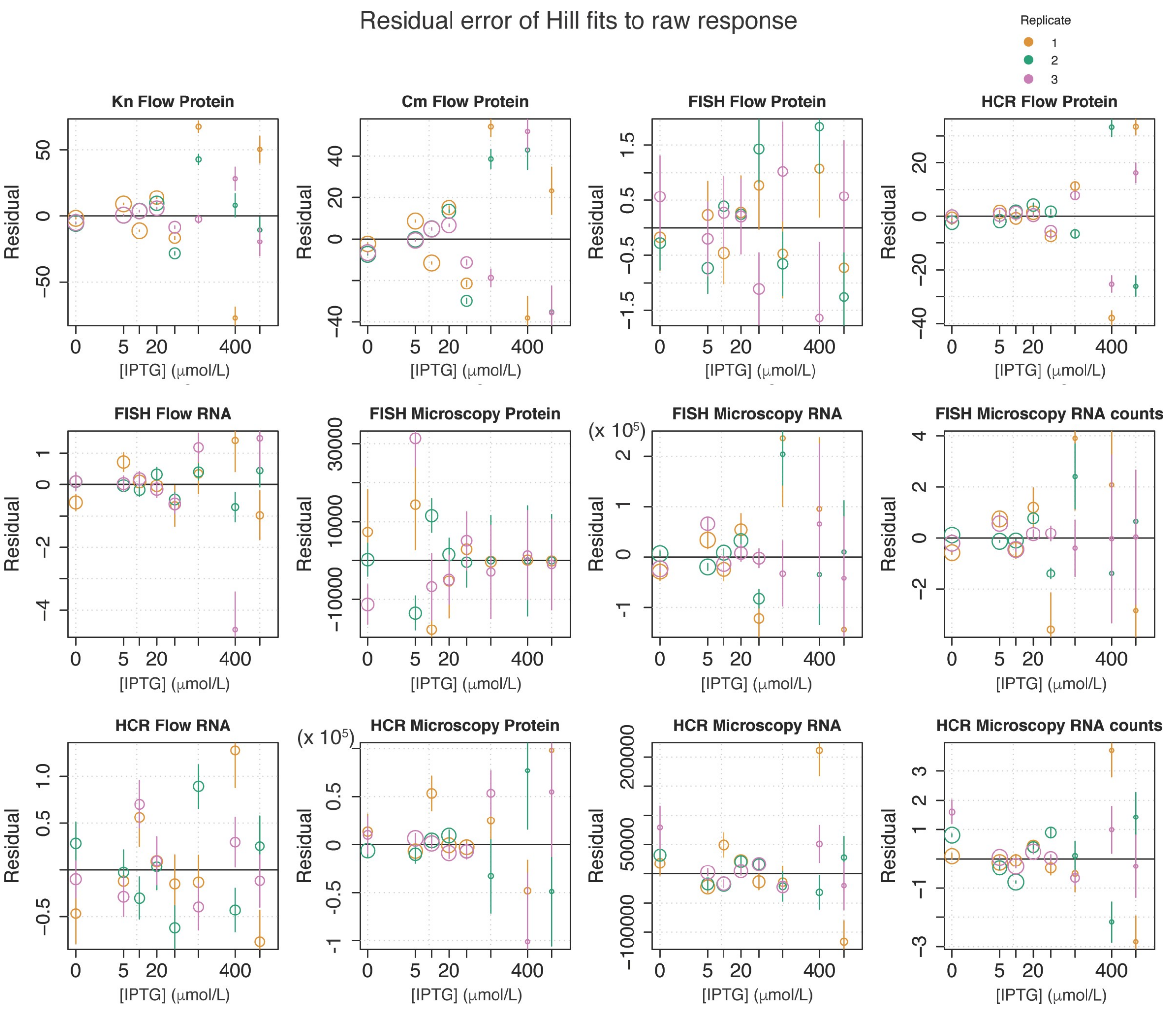


Supplementary Figure 9. Residual error from Hill fits to raw medians. **Residual error from Hill fits to raw medians is plotted for each method.The size of each circle is proportional to its weight in the fit, determined by its inverse variance. Error bars indicate 95 % confidence intervals of each median as determined by bootstrapping using 1,000 iterations. Color indicates biological replicate 1 (orange), replicate 2 (green), and replicate 3 (purple).**

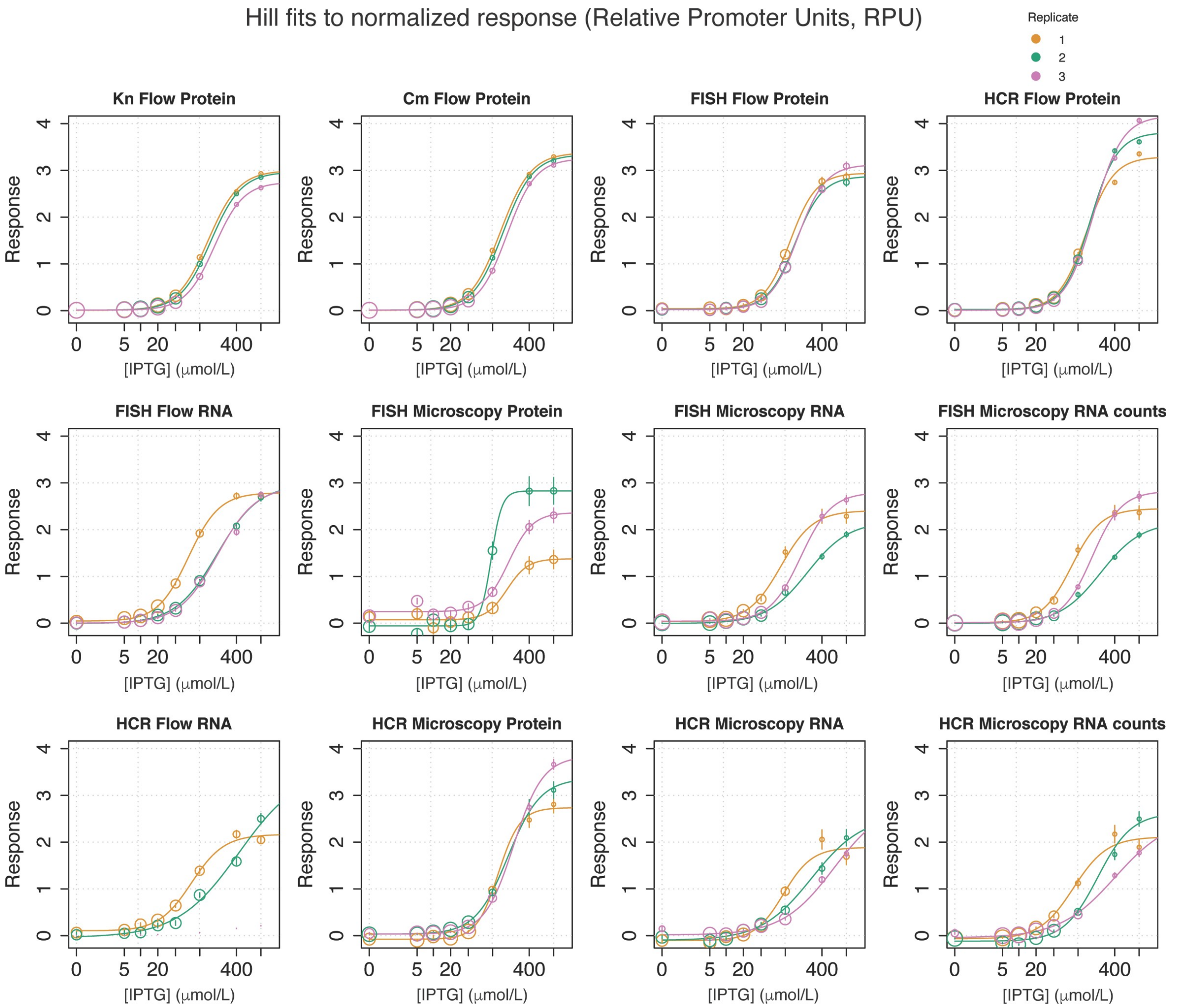


Supplementary Figure 10. Cellular response was quantitatively parameterized using Hill functions fit to RPU-normalized medians. For all 12 measurement methods, the raw medians (circles) were fit to a Hill function (lines) using weighted nonlinear least squares.The size of each circle is proportional to its weight in the fit, determined by its inverse variance. Error bars indicate 95 % confidence intervals of each median as determined by bootstrapping using 1,000 iterations. Color indicates biological replicate 1 (orange), replicate 2 (green), and replicate 3 (purple).

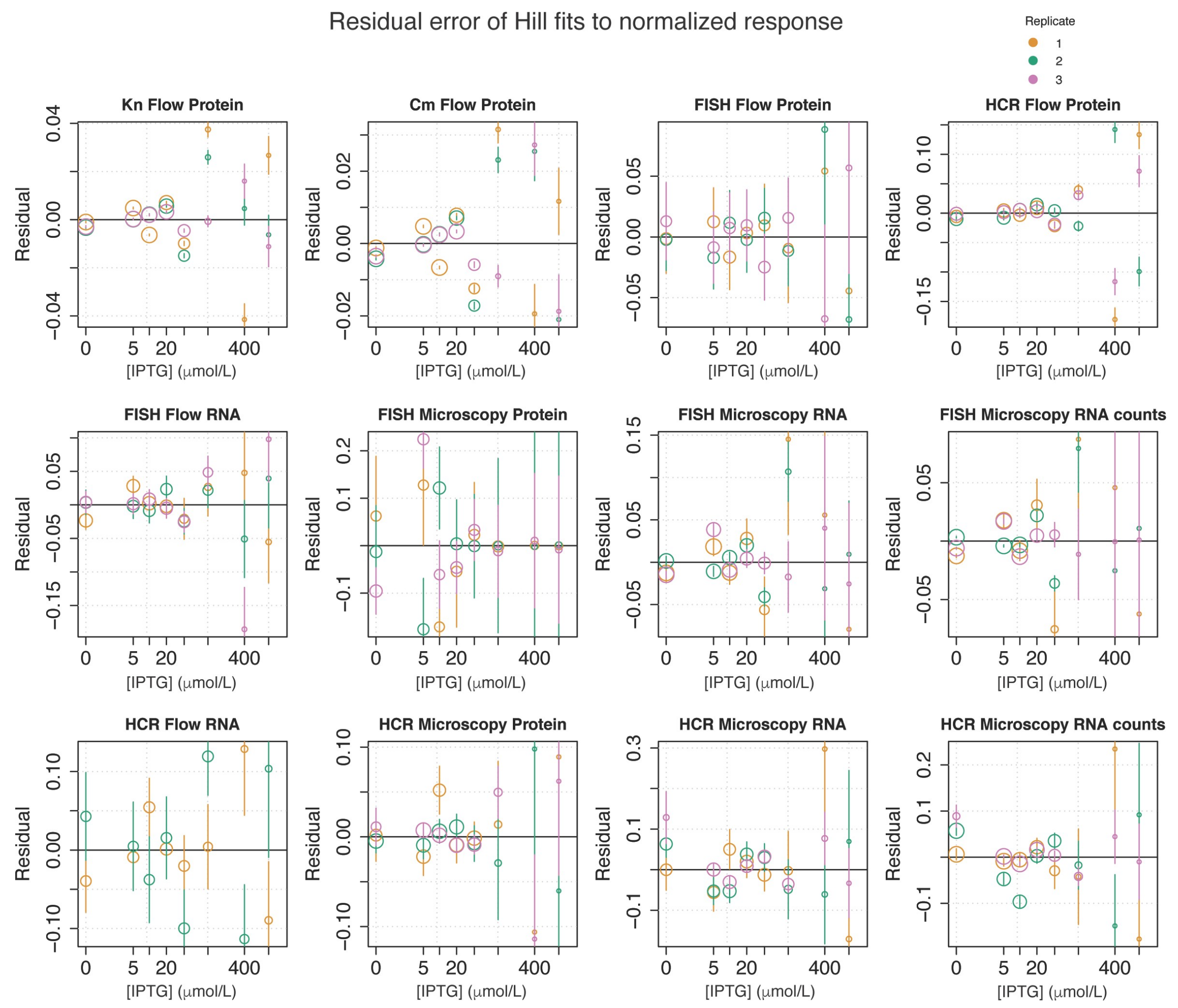


Supplementary Figure 11. Residual error from Hill fits to RPU-normalized medians. Residual error from Hill fits to raw medians is plotted foreach method. The size of each circle is proportional to its weight in the fit, determined by its inverse variance. Error bars indicate 95 % confidence intervals of each median as determined by bootstrapping using 1,000 iterations. Color indicates biological replicate 1 (orange), biological replicate 2 (green), and biological replicate 3 (purple).

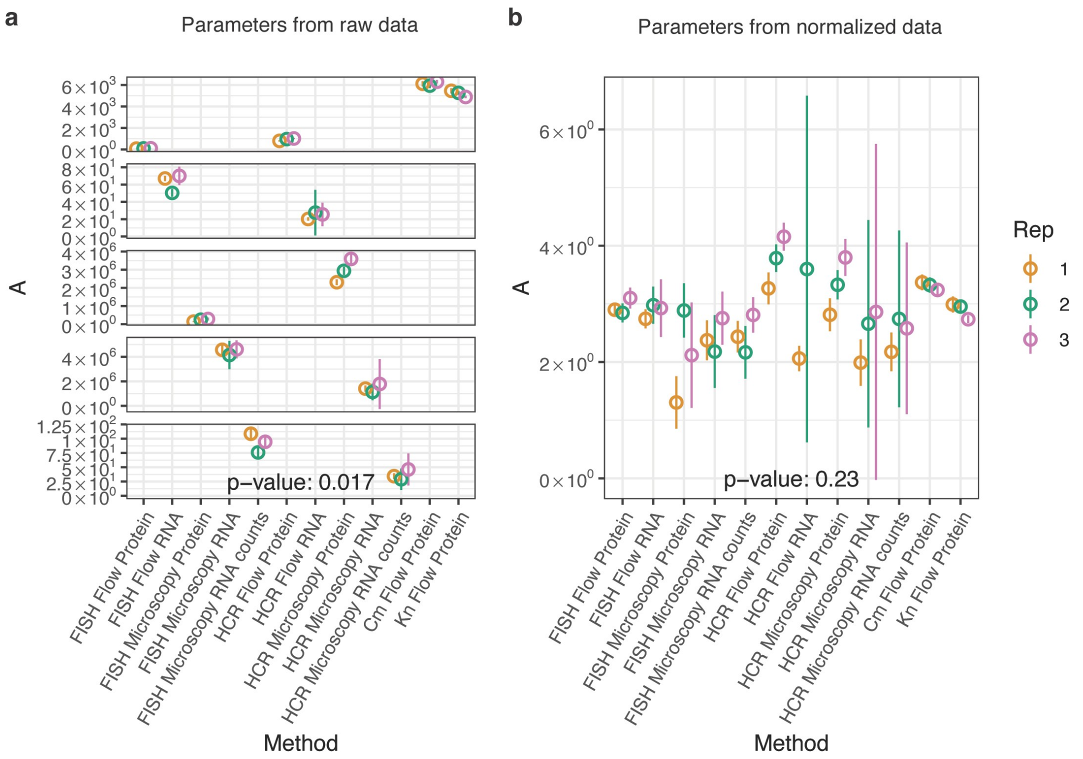


Supplementary Figure 12. Hill parameters for amplitude of raw and RPU-normalized response functions. (a) Parameters estimated from Hill fits to raw medians. Each of the 5 subpanels is for methods that share the same scale of arbitrary units. (b) Parameters estimated from Hill fits to RPU-normalized medians. Error bars represent 95 % confidence intervals from fits, including bootstrapped uncertainty using 1,000 iterations. P-values indicates statistical significance of Friedman test for the null hypothesis that there is no replicate-to-replicate effect. Colors represent biological replicate 1 (orange), biological replicate 2(green), and biological replicate 3 (purple).

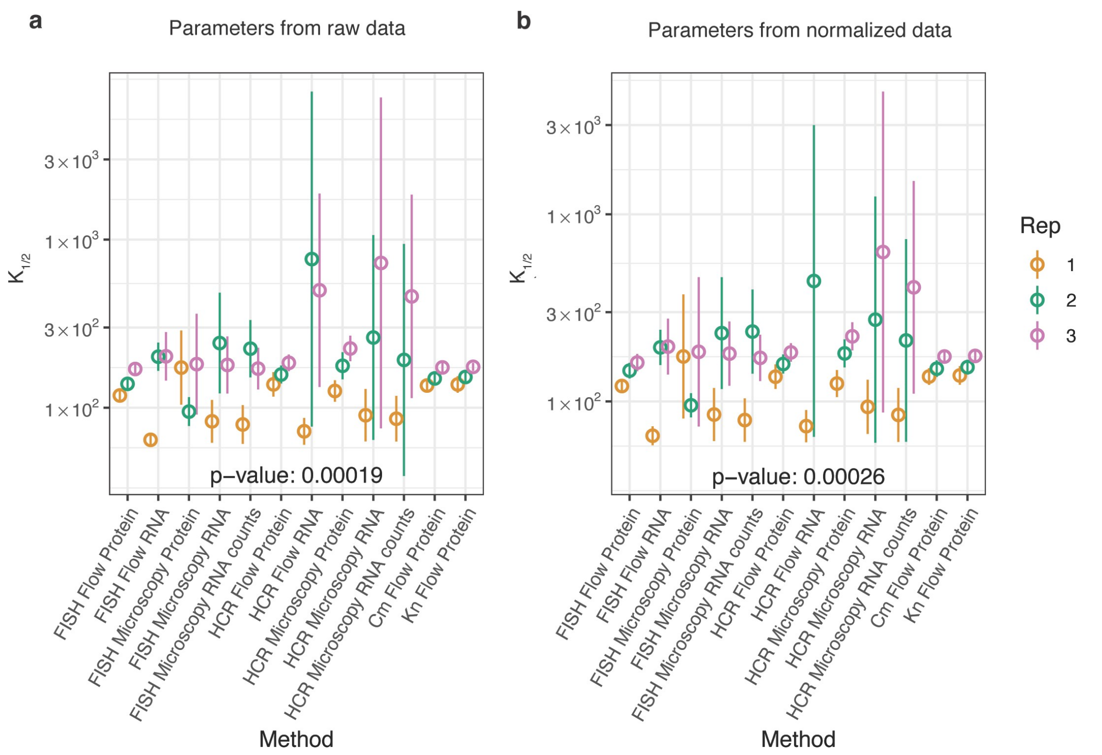


Supplementary Figure 13. Hill parameters for half-maximal induction for raw and RPU-normalized response functions. (a) Parameters estimated from Hill fits to raw medians. (b) Parameters estimated from Hill fits to RPU-normalized medians. Error bars represent 95 % confidence intervals from fits, including bootstrapped uncertainty using 1,000 iterations. P-values indicates statistical significance of Friedman test for the null hypothesis that there is no replicate-to-replicate effect. Colors representbiological replicate 1 (orange), biological replicate 2 (green), and biological replicate 3 (purple).

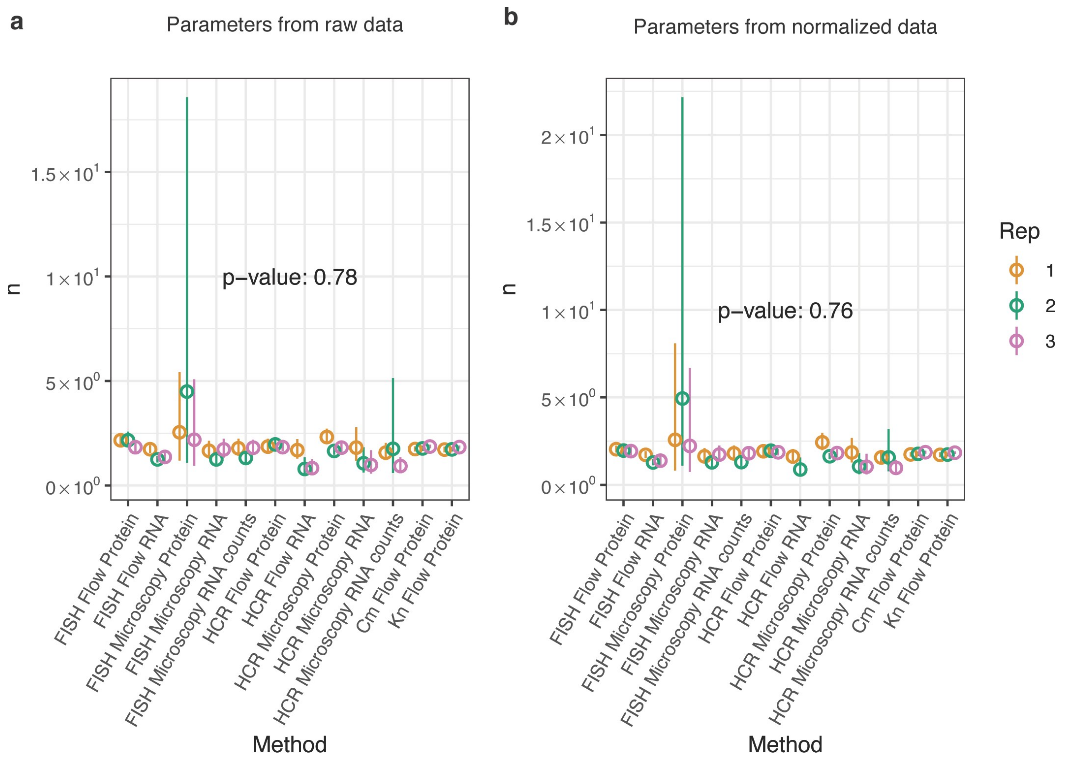


Supplementary Figure 14. Hill parameters for effective cooperativity from raw and RPU-normalized response functions. (a) Parameters estimated from Hill fits to raw medians. (b) Parameters estimated from Hill fits to RPU-normalized medians. Error bars represent 95 % confidence intervals from fits, including bootstrapped uncertainty using 1,000 iterations. P-values indicates statistical significance of Friedman test for the null hypothesis that there is no replicate-to-replicate effect. Colors representbiological replicate 1 (orange), biological replicate 2 (green), and biological replicate 3 (purple).

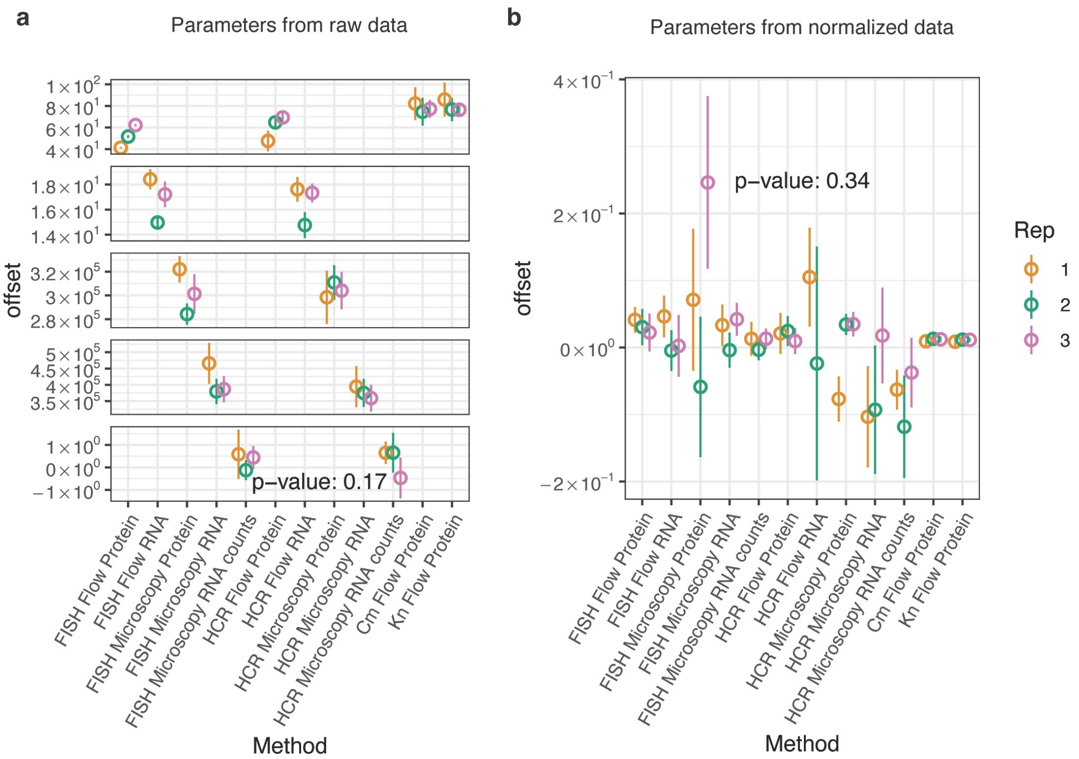


Supplementary Figure 15. Hill parameters for offset from raw and RPU-normalized response functions. (a) Parameters estimated from Hill fits to raw medians. Each of the 5 subpanels is for methods that share the same scale of arbitrary units. P-value in bottom panel was calculated using all 5 panels, as described below. (b) Parameters estimated from Hill fits to RPU-normalized medians.

Error bars represent 95 % confidence intervals from fits, including bootstrapped uncertainty using 1,000 iterations. P-values indicates statistical significance of Friedman test for the null hypothesis that there is no replicate-to-replicate effect, using rankings of replicates within each of the 12 methods. Colors represent biological replicate 1 (orange), biological replicate 2 (green), and biological replicate 3 (purple).

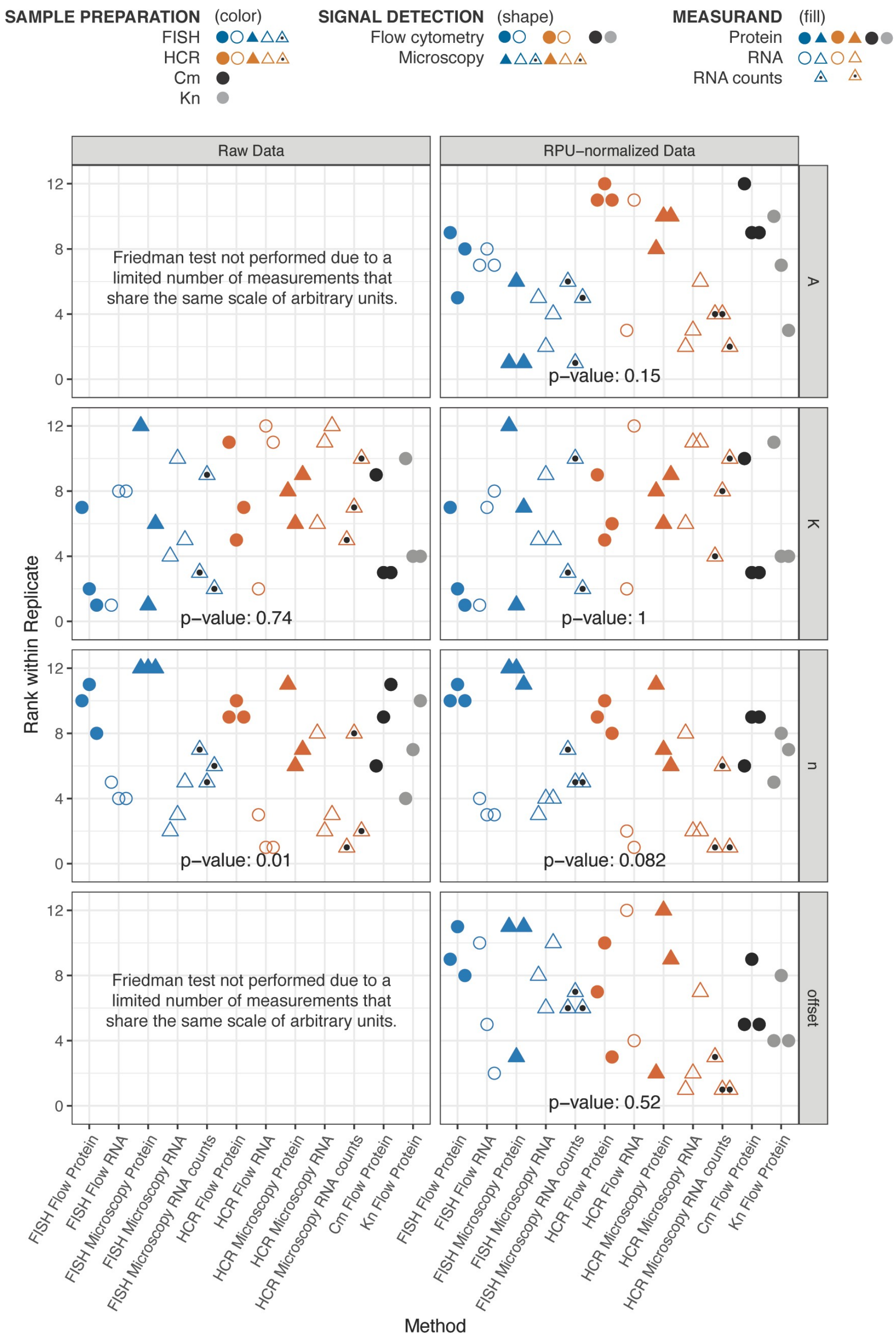


Supplementary Figure 16. Friedman test for relative bias between methods. Within each replicate, each of the four Hill parameters estimatedusing each of the twelve methods were ranked in order from lowest to highest. A random ranking of methods was used as the null hypothesis. For all replicates, p-values shown within each plot indicate the statistical significance of divergence from this null hypothesis. Lower p-values indicate a higher probability of relative bias between methods.

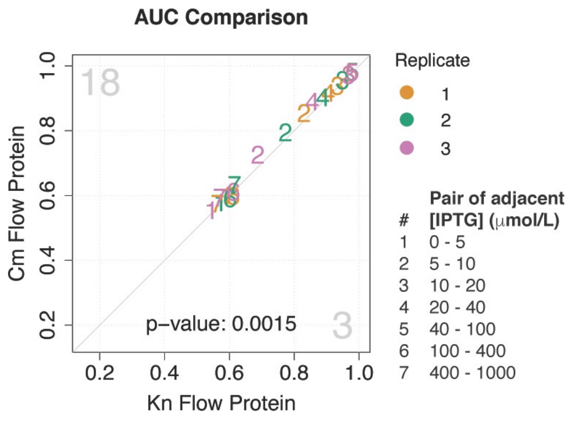


Supplementary Figure 17. Effect of antibiotic treatment on flow cytometry measurement of fluorescent protein prior to *in situ* hybridization.Diagonal line indicates perfect agreement between methods. Measurement performance is attributed to sample preparation by comparing measurements that differ in antibiotic treatment prior to *in situ* hybridization. Aside from this difference in sample preparation, these methods share consistent processes for signal detection, and measurand. Resolvability is quantitatively attributed to sample preparation by comparing AUC profiles. Gray numbers in each corner indicate how many AUC values were larger for one method compared to the equivalent AUC for the other method. Numbers within scatter plot indicate which pair of adjacent stimulus concentrations are used to calculate AUC. Color is used to represent biological replicate 1 (orange), biological replicate 2 (green), and biological replicate 3 (purple). The two-sided p-value for a sign test is shown within the plot.

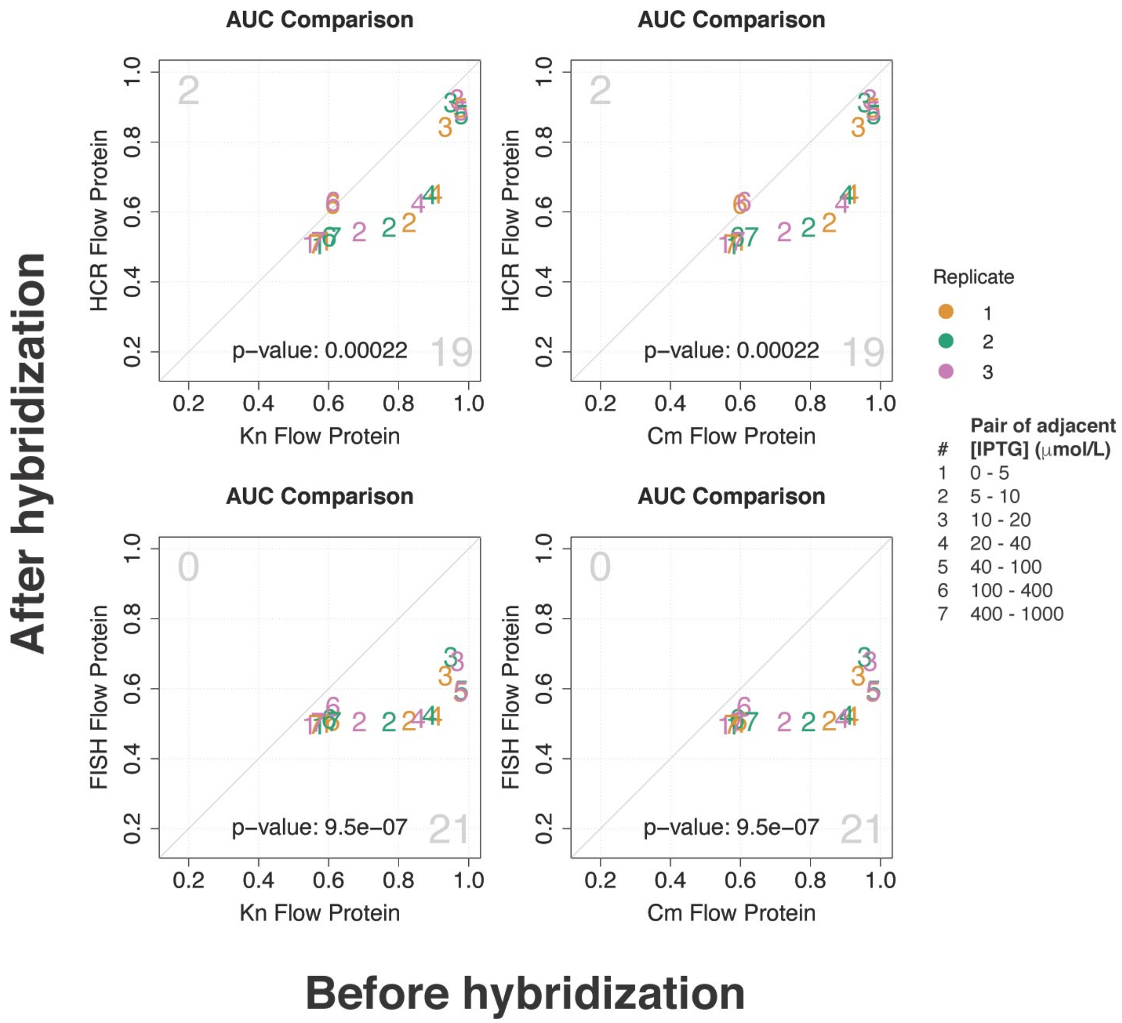


Supplementary Figure 18. Comparison of flow cytometry detection of fluorescent protein before versus after *in situ* hybridization. Measurement performance is attributed to sample preparation by comparing measurements that differ with regard to whether they were measured before or after *in situ* hybridization. Aside from this difference in sample preparation, these methods share consistent measurement steps for signal detection (flow cytometry), and measurand (protein). Resolvabilityis quantitatively attributed to sample preparation by comparing AUC profiles. Diagonal lines indicate perfect agreement between methods. Gray numbers in each corner indicate how many AUC values were larger for one method compared to the equivalent AUC for the other method. Colored umbers within each scatter plot indicate which pair of adjacent stimulusconcentrations are used to calculate AUC. Color is used to represent biological replicate 1 (orange), biological replicate 2 (green), and biological replicate 3 (purple). The two-sided p-values for a sign test are shown within each plot.

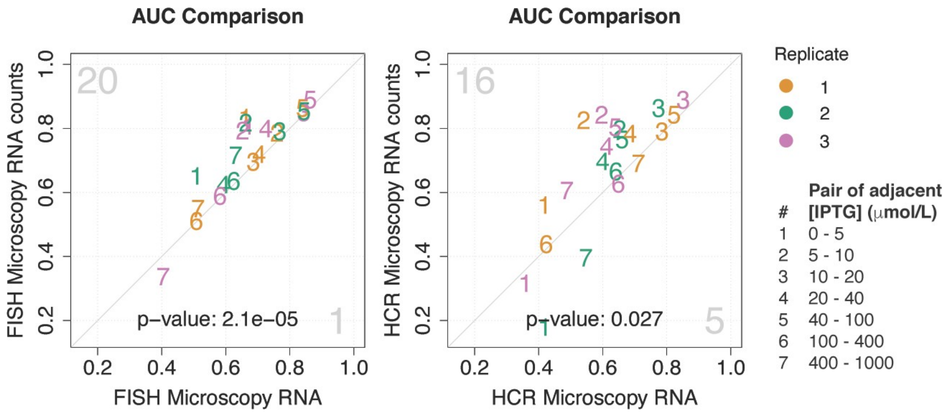


Supplementary Figure 19. Effect of calibrating whole-cell RNA fluorescence intensity to estimate RNA counts. Measurement performance is attributed to measurand by comparing measurements of whole-cell RNA fluorescence versus estimated RNA counts per cellfollowing calibration of microscopy data. Aside from this difference in measurand, these pairwise comparisons share consistent steps for sample preparation, and signal detection (microscopy). Resolvability is quantitatively attributed to measurand by comparing AUC profiles between whole-cell RNA fluorescence and estimated RNA counts per cell for FISH (left) and HCR (right). In each plot, diagonal lines indicate perfect agreement between methods. Gray numbers in each corner indicate how many AUC values were larger for one method compared to the equivalent AUC for the other method. Colored numbers within each scatter plot indicate which pair adjacent stimulus concentrations are used to calculate AUC. Color is used to represent biological replicate 1 (orange), biological replicate 2 (green), and biological replicate 3 (purple). The two-sided p-values for a sign test are shown within each plot.

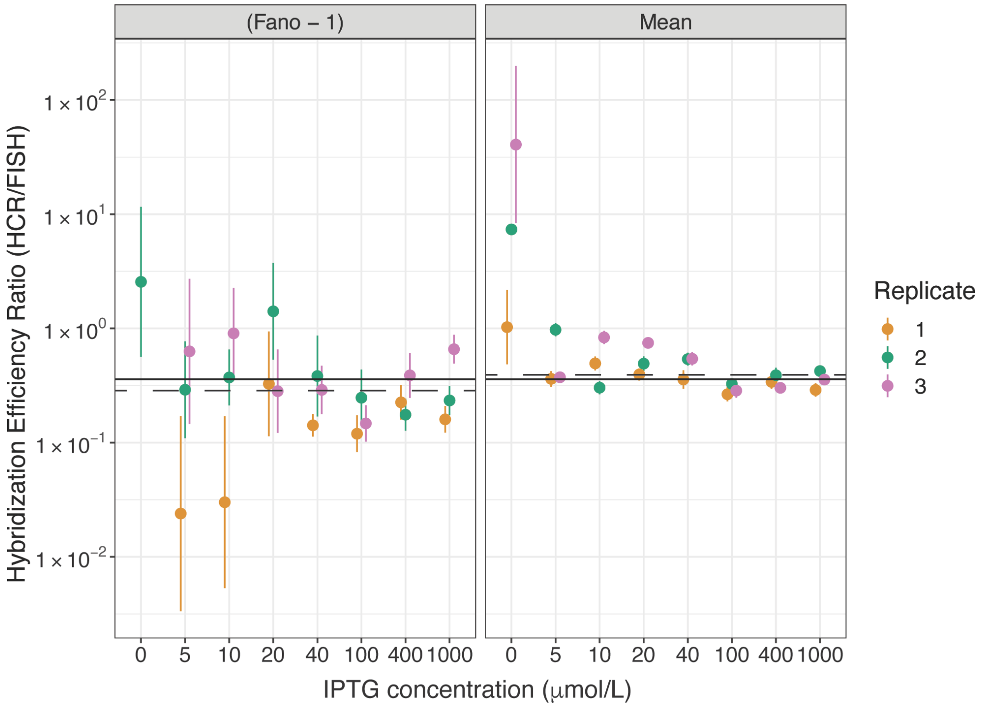


Supplementary Figure 20. Bias between FISH and HCR is consistent with a relative difference in hybridization efficiency. Estimated hybridization efficiency ratios based on ratio of HCR to FISH for (Fano – 1) (left), and based on ratio of HCR to FISH of mean RNA count per cell (right). Horizontal dashed black lines depict the respective median hybridization ratios for point estimatesbased on each type of sample statistic. Horizontal solid black line depicts the overall median hybridization ratio of point estimates from both sample statistic types, 0.359, which was used as our estimated hybridization efficiency ratio of HCR to FISH.

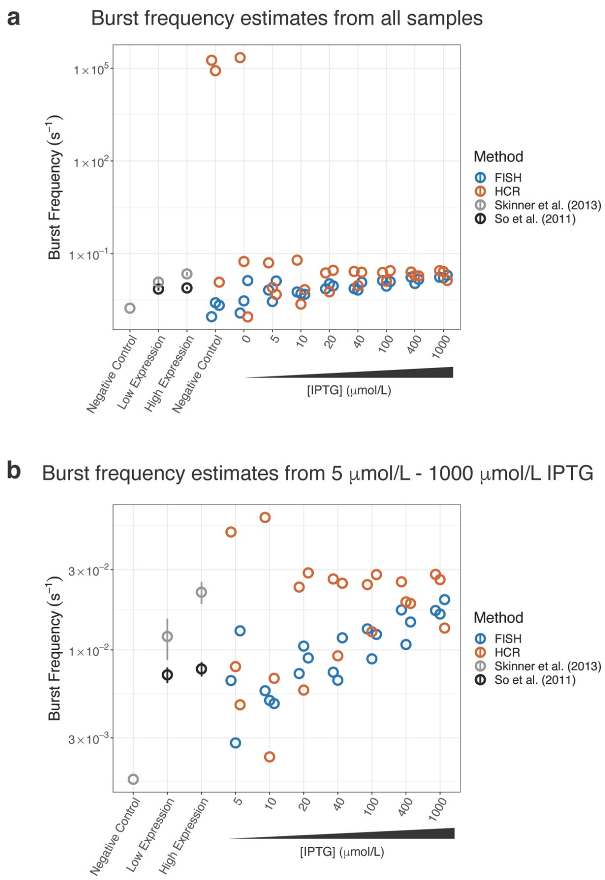


Supplementary Figure 21. Estimates of burst frequency. (a) Estimates of burst frequency are shown for all samples in this study, for both FISH (blue circles) and HCR (red circles). Also shown are estimates of burst frequency from previous studies using FISH for single-transcript detection in *E. coli* (gray and black circles). Error bars for data from previous studies represent uncertaintyas estimated in those studies. (b) Same as (a), except that samples in this study are only shown from 5 μmol/L to 1000 μmol/L IPTG for easier comparison.

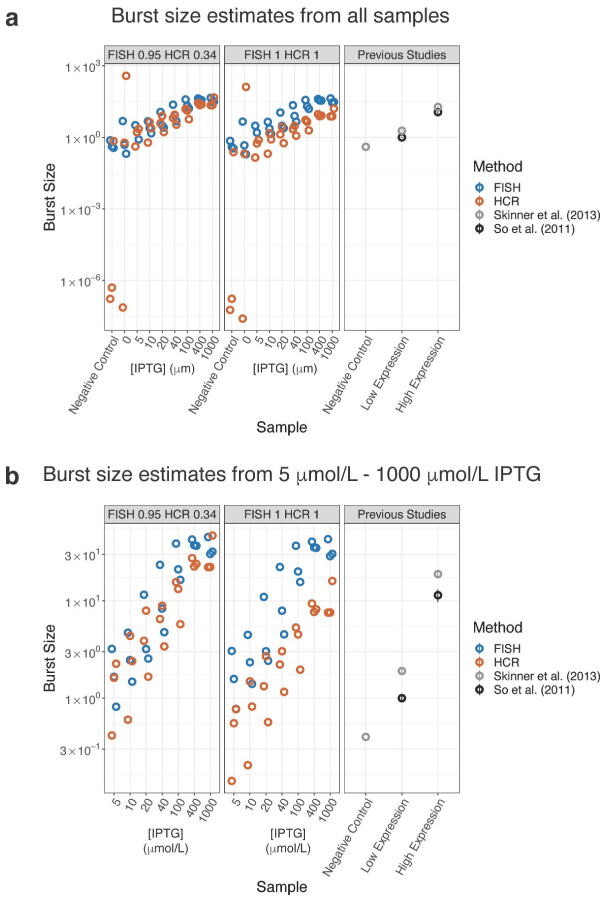


Supplementary Figure 22. Estimates of burst size. (a) Estimates of burst size are shown for all samples in this study, for both FISH (blue circles) and HCR (red circles). *Left:* Estimates of burst size assuming hybridization efficiencies of 0.95 for FISH and 0.34 for HCR. *Middle:* Estimates of burst size for FISH and HCR without any adjustment for hybridization efficiency. *Right:* Estimates of burst size from previous studies using FISH for single-transcript detection in *E.coli* (gray and black circles)[1,2]. Error bars fordata from previous studies represent uncertainty as estimated in those studies. (b) Same as (a), except that samples in this study are only shown from 5 μmol/L to 1000 μmol/L IPTG for easier comparison.

# Supplementary Tables

## Supplementary Table 1. Growth protocol

Growth protocol is presented as recommended by the Minimum Information Standard for Engineering Organism Experiments (MIEO)[3].

| MIEO Category | Factor | Level | |
|---|---|---|---|
| **Media components** | Potassium phosphate | 3 g/L | |
| | Disodium phosphate | 6.78 g/L | |
| | Sodium chloride | 0.5 g/L | |
| | Ammonium chloride | 1.0 g/L | |
| | D-glucose | 4.0 g/L | |
| | Casamino acids | 2.0 g/L | |
| | Calcium chloride | 0.1 g/L | |
| | Magnesium sulfate | 0.493 g/L | |
| | Vitamin B1 (Thiamine) | 0.34 g/L | |
| | Water | DI water (18 MΩ-cm) | |
| **Media properties** | pH | 7.4 | |
| | Osmolality | Not measured | |
| | | *Overnight growth* | *Outgrowth* |
| **Container geometry** | Type | “culture” tube | “Falcon” tube |
| | Container shape | Round | Round |
| | Container bottom | Round | Conical |
| | Container volume | 14 mL | 50 mL |
| | Fill volume | 14 % (2 mL) | 40 % (20 mL) |
| | Cover | Snap cap | Screw cap, ¼ turn loosened |
| **Container shaking** | Shaking speed | 200 rpm | 200 rpm |
| | Shaking diameter | 2.5 cm | 2.5 cm |
| | Shaking mode | Orbital | Orbital |
| **Time** | Growth time | 16.0 hours | 3.5 hours |
| **Environment** | Temperature | 37 °C | 37 °C |
| | | | |
| **Selective agents** | Antibiotic type | Kanamycin | Kanamycin |
| | Antibiotic concentration | 50 μg/ml | 50 μg/ml |
| **Inoculum** | Type | Single colony | Culture |
| | Concentration at inoculation | N/A | Optical density at 600 nm = 0.22 ± 0.01* |
| | Age of inoculum at inoculation | N/A | |
| | | | |
| **Inducers** | Plasmid | [IPTG] (μmol/L) | [IPTG] (μmol/L) |
| Culture 1 | pAN1201 | 0 | 0 |
| Culture 2 | pAN1717 | 0 | 0 |
| Culture 3 | pAN1818 | 0 | 0 |
| Culture 4 | pAN1818 | | 5 |
| Culture 5 | pAN1818 | | 10 |
| Culture 6 | pAN1818 | | 20 |
| Culture 7 | pAN1818 | | 40 |
| Culture 8 | pAN1818 | | 100 |
| Culture 9 | pAN1818 | | 400 |
| Culture 10 | pAN1818 | | 1000 |
| | | | |

*Average ± standard deviation, all samples and replicates.

## Supplementary Table 2. DNA sequences used in this work.

**Genetic part sequences**

| Part name | Type | DNA sequence |
|---|---|---|
| BBa_J23101 | promoter | TTTACAGCTAGCTCAGTCCTAGGTATTATGCTAGC |
| Ptac | promoter | AACGATCGTTGGCTGTGTTGACAATTAATCATCGGCTCGTATAATGTGTGGAATTGTGAGCG<br>CTCACAATT |
| RiboJ | insulator | AGCTGTCACCGGATGTGCTTTCCGGTCTGATGAGTCCGTGAGGACGAAACAGCCTCTACAAA<br>TAATTTTGTTTAA |
| eyfp | gene | ATGGTGAGCAAGGGCGAGGAGCTGTTCACCGGGGTGGTGCCCATCCTGGTCGAGCTGGACGG<br>CGACGTAAACGGCCACAAGTTCAGCGTGTCCGGCGAGGGCGAGGGCGATGCCACCTACGGCA<br>AGCTGACCCTGAAGTTCATCTGCACCACAGGCAAGCTGCCCGTGCCCTGGCCCACCCTCGTG<br>ACCACCTTCGGCTACGGCCTGCAATGCTTCGCCCGCTACCCCGACCACATGAAGCTGCACGA<br>CTTCTTCAAGTCCGCCATGCCCGAAGGCTACGTCCAGGAGCGCACCATCTTCTTCAAGGACG<br>ACGGCAACTACAAGACCCGCGCCGAGGTGAAGTTCGAGGGCGACACCCTGGTGAACCGCATC<br>GAGCTGAAGGGCATCGACTTCAAGGAGGACGGCAACATCCTGGGGCACAAGCTGGAGTACAA<br>CTACAACAGCCACAACGTCTATATCATGGCCGACAAGCAGAAGAACGGCATCAAGGTGAACT<br>TCAAGATCCGCCACAACATCGAGGACGGCAGCGTGCAGCTCGCCGACCACTACCAGCAGAAC<br>ACCCCAATCGGCGACGGCCCCGTGCTGCTGCCCGACAACCACTACCTTAGCTACCAGTCCGC<br>CCTGAGCAAAGACCCCAACGAGAAGCGCGATCACATGGTCCTGCTGGAGTTCGTGACCGCCG<br>CCGGGATCACTCTCGGCATGGACGAGCTGTACAAGTAA |
| LacI | gene | ATGAAACCAGTAACGTTATACGATGTCGCAGAGTATGCCGGTGTCTCTTATCAGACCGTTTC<br>CCGCGTGGTGAACCAGGCCAGCCACGTTTCTGCGAAAACGCGGGAAAAAGTGGAAGCGGCGA<br>TGGCGGAGCTGAATTACATTCCCAACCGCGTGGCACAACAACTGGCGGGCAAACAGTCGTTG<br>CTGATTGGCGTTGCCACCTCCAGTCTGGCCCTGCACGCGCCGTCGCAAATTGTCGCGGCGAT<br>TAAATCTCGCGCCGATCAACTGGGTGCCAGCGTGGTGGTGTCGATGGTAGAACGAAGCGGCG<br>TCGAAGCCTGTAAAGCGGCGGTGCACAATCTTCTCGCGCAACGCGTCAGTGGGCTGATCATT<br>AACTATCCGCTGGATGACCAGGATGCCATTGCTGTGGAAGCTGCCTGCACTAATGTTCCGGC<br>GTTATTTCTTGATGTCTCTGACCAGACACCCATCAACAGTATTATTTTCTCCCATGAGGACG<br>GTACGCGACTGGGCGTGGAGCATCTGGTCGCATTGGGTCACCAGCAAATCGCGCTGTTAGCG<br>GGCCCATTAAGTTCTGTCTCGGCGCGTCTGCGTCTGGCTGGCTGGCATAAATATCTCACTCG<br>CAATCAAATTCAGCCGATAGCGGAACGGGAAGGCGACTGGAGTGCCATGTCCGGTTTTCAAC<br>AAACCATGCAAATGCTGAATGAGGGCATCGTTCCCACTGCGATGCTGGTTGCCAACGATCAG<br>ATGGCGCTGGGCGCAATGCGCGCCATTACCGAGTCCGGGCTGCGCGTTGGTGCGGATATCTC<br>GGTAGTGGGATACGACGATACCGAAGATAGCTCATGTTATATCCCGCCGTTAACCACC |
| L3S3SP21 | terminator | CTCGGTACCAAATTCCAGAAAAGAGGCCTCCCGAAAGGGGGGCCTTTTTTCGTTTTGGTCC |

## Supplementary Table 3: FISH and HCR Probes used in this work

**FISH Probes**

| Probe name | Size (nt) | % GC | DNA sequence |
|---|---|---|---|
| eyfp1 | 20 | 60% | TCCTCGCCCTTGCTCACCAT |
| eyfp2 | 20 | 50% | GCTGAACTTGTGGCCGTTTA |
| eyfp3 | 20 | 65% | CAGGGTCAGCTTGCCGTAGG |
| eyfp4 | 20 | 55% | TGCCTGTGGTGCAGATGAAC |
| eyfp5 | 20 | 60% | GTAGCCGAAGGTGGTCACGA |
| eyfp6 | 20 | 60% | TAGCGGGCGAAGCATTGCAG |
| eyfp7 | 20 | 60% | GTGCAGCTTCATGTGGTCGG |
| eyfp8 | 20 | 55% | GCATGGCGGACTTGAAGAAG |
| eyfp9 | 20 | 65% | CGCTCCTGGACGTAGCCTTC |
| eyfp10 | 20 | 50% | GTCGTCCTTGAAGAAGATGG |
| eyfp11 | 20 | 65% | CGGCGCGGGTCTTGTAGTTG |
| eyfp12 | 20 | 60% | GTGTCGCCCTCGAACTTCAC |
| eyfp13 | 20 | 55% | TTCAGCTCGATGCGGTTCAC |
| eyfp14 | 20 | 55% | CGTCCTCCTTGAAGTCGATG |
| eyfp15 | 20 | 55% | AGCTTGTGCCCCAGGATGTT |
| eyfp16 | 20 | 45% | GTGGCTGTTGTAGTTGTACT |
| eyfp17 | 20 | 50% | TGTCGGCCATGATATAGACG |
| eyfp18 | 20 | 50% | ACCTTGATGCCGTTCTTCTG |
| eyfp19 | 20 | 50% | TGTTGTGGCGGATCTTGAAG |
| eyfp20 | 20 | 55% | TGTTCTGCTGGTAGTGGTCG |
| eyfp21 | 20 | 55% | CTAAGGTAGTGGTTGTCGGG |
| eyfp22 | 20 | 65% | CTTTGCTCAGGGCGGACTGG |
| eyfp23 | 20 | 60% | TGATCGCGCTTCTCGTTGGG |
| eyfp24 | 20 | 60% | CACGAACTCCAGCAGGACCA |
| eyfp25 | 20 | 45% | TACTTGTACAGCTCGTCCAT |

**HCR Probes**

| Probe name | Size (nt) | %GC | Targeted mRNA subsequence |
|---|---|---|---|
| B1 P1 | 52 | 65 | ATGGTGAGCAAGGGCGAGGAGCTGTTCACCGGGGTGGTGCCCATCCTGGTCG |
| B1 P2 | 52 | 67 | CTGGACGGCGACGTAAACGGCCACAAGTTCAGCGTGTCCGGCGAGGGCGAGG |
| B1 P3 | 52 | 58 | GATGCCACCTACGGCAAGCTGACCCTGAAGTTCATCTGCACCACAGGCAAGC |
| B1 P4 | 52 | 69 | CCCGTGCCCTGGCCCACCCTCGTGACCACCTTCGGCTACGGCCTGCAATGCT |
| B1 P5 | 52 | 62 | GCCCGCTACCCCGACCACATGAAGCTGCACGACTTCTTCAAGTCCGCCATGC |
| B1 P6 | 52 | 56 | GAAGGCTACGTCCAGGAGCGCACCATCTTCTTCAAGGACGACGGCAACTACA |
| B1 P7 | 52 | 67 | ACCCGCGCCGAGGTGAAGTTCGAGGGCGACACCCTGGTGAACCGCATCGAGC |
| B1 P8 | 52 | 58 | AAGGGCATCGACTTCAAGGAGGACGGCAACATCCTGGGGCACAAGCTGGAGT |
| B1 P9 | 52 | 48 | AACTACAACAGCCACAACGTCTATATCATGGCCGACAAGCAGAAGAACGGCA |
| B1 P10 | 52 | 58 | AAGGTGAACTTCAAGATCCGCCACAACATCGAGGACGGCAGCGTGCAGCTCG |
| B1 P11 | 52 | 69 | GACCACTACCAGCAGAACACCCCAATCGGCGACGGCCCCGTGCTGCTGCCCG |
| B1 P12 | 52 | 56 | AACCACTACCTTAGCTACCAGTCCGCCCTGAGCAAAGACCCCAACGAGAAGC |
| B1 P13 | 52 | 60 | GTTCGTGACCGCCGCCGGGATCACTCTCGGCATGGACGAGCTGTACAAGTAA |

**HCR B1 Amplifier (Hairpin) Sequences**

| Module | DNA sequence |
|---|---|
| I1 | GAGGAGGGCAGCAAACGGGAAGAGTCTTCCTTTACG |
| I2 | GCATTCTTTCTTGAGGAGGGCAGCAAACGGGAAGAG |
| H1 | CGTAAAGGAAGACTCTTCCCGTTTGCTGCCCTCCTCGCATTCTTTCTTGAGGAGGGCAGCAAACGGGAAGAG /C9-TAMRA-3'/ |
| H2 | /5'-TAMRA-C12/ GAGGAGGGCAGCAAACGGGAAGAGTCTTCCTTTACGCTCTTCCCGTTTGCTGCCCTCCTCAAGAAAGAATGC |

## Supplementary Table 4: Number of cells for each measurement

| Method | Sample | [IPTG] (µmol/L) | Number of cells Replicate 1 | Replicate 2 | Replicate 3 |
|---|---|---|---|---|---|
| FISH Flow Protein<br>FISH Flow RNA | pAN1201 | 0 | 129632 | 45784 | 61285 |
| | pAN1717 | 0 | 78361 | 47261 | 75294 |
| | pAN1818 | 0 | 73376 | 126498 | 56793 |
| | | 5 | 67421 | 140014 | 72530 |
| | | 10 | 100625 | 88795 | 71563 |
| | | 20 | 58481 | 84760 | 71786 |
| | | 40 | 47244 | 80109 | 67900 |
| | | 100 | 58199 | 100517 | 46763 |
| | | 400 | 72072 | 72905 | 34238 |
| | | 1000 | 79901 | 85273 | 62734 |
| FISH Microscopy Protein<br>FISH Microscopy RNA<br>FISH Microscopy RNA counts | pAN1201 | 0 | 1812 | 2180 | 2715 |
| | pAN1717 | 0 | 1095 | 3566 | 2549 |
| | pAN1818 | 0 | 584 | 2877 | 2614 |
| | | 5 | 950 | 2181 | 1500 |
| | | 10 | 839 | 2977 | 1217 |
| | | 20 | 893 | 3094 | 1845 |
| | | 40 | 1042 | 1362 | 1508 |
| | | 100 | 1504 | 1156 | 1041 |
| | | 400 | 1476 | 1360 | 1221 |
| | | 1000 | 1548 | 2202 | 2143 |
| HCR Flow Protein<br>HCR Flow RNA | pAN1201 | 0 | 80535 | 53372 | 96100 |
| | pAN1717 | 0 | 59702 | 85483 | 86411* |
| | pAN1818 | 0 | 44188 | 70429 | 115004 |
| | | 5 | 66890 | 72191 | 98063 |
| | | 10 | 52344 | 72910 | 90639 |
| | | 20 | 86539 | 64971 | 117528 |
| | | 40 | 64574 | 60900 | 86681* |
| | | 100 | 81198 | 82340 | 90516 |
| | | 400 | 58659 | 94663 | 95216 |
| | | 1000 | 80894 | 71423 | 94915 |
| HCR Microscopy Protein<br>HCR Microscopy RNA<br>HCR Microscopy RNA counts | pAN1201 | 0 | 165 | 746 | 1326 |
| | pAN1717 | 0 | 405 | 733 | 2560 |
| | pAN1818 | 0 | 299 | 1939 | 110 |
| | | 5 | 899 | 916 | 2115 |
| | | 10 | 191 | 1482 | 1752 |
| | | 20 | 1506 | 1223 | 2820 |
| | | 40 | 2786 | 2148 | 2972 |
| | | 100 | 1691 | 768 | 1678 |
| | | 400 | 2595 | 2043 | 1365 |
| | | 1000 | 2134 | 2017 | 2410 |

*HCR Flow RNA was excluded for these samples due to a flow cytometer malfunction.

(continued on next page)

(Supplementary Table 4 continued)

| Method | Sample | [IPTG] (μmol/L) | Number of cells Replicate 1 | Replicate 2 | Replicate 3 |
|---|---|---|---|---|---|
| Cm Flow Protein | pAN1201 | 0 | 217453 | 192743 | 197950 |
| | pAN1717 | 0 | 241520 | 191203 | 127242 |
| | pAN1818 | 0 | 243881 | 206080 | 190298 |
| | | 5 | 209810 | 201452 | 160804 |
| | | 10 | 214210 | 220835 | 182677 |
| | | 20 | 227961 | 205403 | 189716 |
| | | 40 | 244653 | 210465 | 178098 |
| | | 100 | 220312 | 196286 | 157032 |
| | | 400 | 203255 | 215339 | 174872 |
| | | 1000 | 219410 | 202068 | 152674 |
| Kn Flow Protein | pAN1201 | 0 | 191301 | 196628 | 195482 |
| | pAN1717 | 0 | 245090 | 206573 | 175496 |
| | pAN1818 | 0 | 216514 | 201026 | 219477 |
| | | 5 | 197570 | 210316 | 200951 |
| | | 10 | 197476 | 213194 | 205087 |
| | | 20 | 220000 | 224146 | 199588 |
| | | 40 | 229563 | 202373 | 194451 |
| | | 100 | 224271 | 208902 | 194393 |
| | | 400 | 219319 | 213270 | 190093 |
| | | 1000 | 231717 | 219370 | 164505 |

# Supplementary Notes

## Supplementary Note 1: General protocol for Bias and Resolvability Attribution using Split Samples (BRASS)

To implement BRASS for comparing any measurement process, consider the following step-by-step protocol.

1. Define performance test(s) to assess measurement performance. Ideally, performance tests are quantitative to enable statistical comparison between different measurements, but qualitative comparisons can be used as well. You can choose more than one.

    a. Example performance tests (T)
    In this study, we were interested in comparing
    $T_1$ – Resolvability was assessed using Area under the ROC Curve (AUC)
    $T_2$ – Relative bias between single-cell measurements was assessed by fitting data to models of cellular response, and comparing the parameters estimated using different methods
        1) Dose-response estimated from Hill equation
        2) Transcription kinetics estimated from burst size and frequency

2. Identify measurement steps of interest which can be evaluated using the performance tests defined in step 1. Identify steps by listing out components of each measurement pipeline, for example using an Ishikawa "Fishbone" diagram.

    a. Examples for Sample Preparation (P)
    In this study, we were interested in comparing two different sample preparation strategies for labeling RNA (FISH and HCR). We were also interested in comparing different antibiotic treatments prior to flow cytometry detection of protein (Kn versus Cm).
    $P_1$ – FISH
    $P_2$ – HCR
    $P_3$ – Kn
    $P_4$ – Cm

    b. Examples for Signal Detection (D)
    In this study, we compared two different ways of detecting single-cell fluorescence (flow cytometry versus microscopy).
    $D_1$ – Flow cytometry
    $D_2$ – Microscopy

    c. Examples for choice of Measurand (M)

In this study, we compared two different physical measurands (RNA versus protein). Measurands can also arise using different analysis processes (RNA whole-cell fluorescence versus estimated RNA counts per cell).
$M_1$ – Protein
$M_2$ – RNA
$M_3$ – RNA counts (estimated counts per cell)

3. Prioritize measurements according to how many can be practically executed in parallel from a single starting sample.

    a. Example for selection of measurements
    Based on exploratory measurements, we found that we needed ~ 0.5 mL of bacterial culture for at least two different preparations of flow cytometry measurements prior to fixation. We also need six mL of bacterial culture for FISH, and another six mL of bacterial culture for HCR to provide enough material for microscopy and flow cytometry following RNA labeling. Additional measurands did not require any additional starting material, since they were detected from within cells, or generated during analysis. So, 12.5 mL of starting culture would be needed in total for all measurements. We chose to grow 20 mL of culture which can easily be performed in a 50 mL Falcon tube, which provided a sufficient quantity of starting sample for all subsequent measurements.

4. Prioritize samples according to what is needed to assess performance, and how many can be practically executed in a single experiment.

    a. Considerations of performance tests for sample selection
        i. Resolvability – a minimum of 2 different levels of response are required to assess a measurement's ability to resolve change in stimulus.
        ii. Calibrating whole-cell fluorescence to estimate single-transcript count – a minimum of 4 concentrations at low induction are needed for estimating the initial slope of the calibration curve[4].
        iii. Parametric evaluations using Hill functions – initial, exploratory experiments suggested that 8 levels of stimulus spanning the dynamic range of response would be sufficient to fit a Hill function.
        iv. Normalization of cellular response to Relative Promoter Units using a living reference material – in addition to 8 levels of stimulus, a negative control (using a plasmid lacking the expression cassette) and a positive control (constitutive expression from J23101) are required for background subtraction and normalization.
        v. The above constraints would be satisfied using a total of 10 samples: 1 negative control, 1 positive control, and 8 different levels of induction.

b. Considerations of experimental constraints for sample selection
   Based on previous experiments, we found that manually preparing and imaging ~20 samples at once was a practical limitation for *in situ* hybridization. So, we chose 10 difference samples including 1 negative control, 1 positive control, and 8 concentrations of induction. This way, after the sample were split for labeling by FISH or HCR, there would be a total of 20 samples to prepare and image (10 for FISH and 10 for HCR). And, the samples would provide the requisite conditions to assess bias in dose-response.

5. Design and execute the experiment including all samples and measurements identified in steps 1-4. Ensure that sufficient reagents are available for the requisite number of replicates, in order to eliminate batch-dependent variability between replicates. In this study, we chose to execute 3 biological replicates.

6. Analyze the data. In this step, various analyses can be performed to generate multiple "measurands". For example, calibrating RNA fluorescence per cell to estimate RNA abundance per cell was performed during analysis, and each of these measurands can lend themselves to a variety of performance tests.

7. To attribute measurement performance to measurement processes, compare performance tests between measurements in a pairwise fashion that keeps all processes the same except for the process of interest.

   Compare 2 different Measurands ($M_1$ vs. $M_2$) by keeping Preparation (P) and Detection (D) constant:
   $(P_x,D_y,M_1)$ vs. $(P_x,D_y,M_2)$

   Compare 2 different Detections ($D_1$ vs. $D_2$) by keeping Preparation (P) and Measurand (M) constant:
   $(P_x,D_1,M_z)$ vs. $(P_x,D_2,M_z)$

   Compare 2 different Preparations ($P_1$ vs. $P_2$) by keeping Detection (D) and Measurand (M) constant:
   $(P_1,D_y,M_z)$ vs. $(P_2,D_y,M_z)$

   More comparisons of the same step can increase confidence in attribution of relative bias. Examples in this study used at least 4 different comparisons of the same step.

   Fractional-factorial design of experiments can be used to more efficiently explore how measurements performance can be attributed to different steps, and how to account for confounding effects between measurement pipelines that differ by multiple steps[3,5].

## Supplementary Note 2: HCR protocol for single-transcript detection in bacteria

STEP 1: Fixation and permeabilization of bacteria in suspension for transcript labeling by *in situ* hybridization (compatible with either HCR or FISH)

1. Pick a single colony of *E. coli* from streaked plate of LB agar with 50 mg/ml Kanamycin and inoculate 2 mL M9 media with Kanamycin at 50 mg/ml. Grow for 16 hours overnight in a 37 °C shaker at 2500 rpm.
2. Inoculate 20 mL of liquid culture for each sample diluting 1:300 overnight culture with same M9+ media.
3. Induce culture samples with IPTG as needed.
4. Incubate in a 37 °C shaker at 2500 rpm until $OD_{600}$ = 0.2 – 0.3 (exponential phase).
5. Remove cultures and place them on ice. At this point, harvested cells can be split for different sample preparations. For example, aliquot 6 mL for HCR and 6 mL for FISH, each in 15 mL conical tubes. Remaining 8 mL culture can be used for flow cytometry.
6. Centrifuge for 10 min at room temperature (~25 °C) at 4000 g.
7. Remove supernatant and re-suspend cells in 750 µL of 1x phosphate-buffered saline (PBS), transfer to 1.5 mL Eppendorf tube.
8. Add 250 µL of 4 % formaldehyde in 1x PBS to and incubate for 16 hours overnight at 4 °C.
9. Centrifuge for 10 min at 4 °C at 4000 g and remove supernatant.
10. Re-suspend cells in 150 µL of 1x PBS.
11. Add 850 mL of 100 % MeOH and incubate at 4 °C for 3.5 hours.

*After Step 1, the protocols for HCR and FISH are different.*

STEP 2: Detection stage (hybridization of HCR probes to target RNA)

1. Transfer 1000 µL of fixed cells into a new 1.5 mL Eppendorf tube.
2. Centrifuge for 5 min at 4°C at 4000 g and remove supernatant.
3. Wash cells with 500 mL of 1x PBST buffer. Centrifuge at 4°C at 4000g for 5 min, remove supernatant.
4. Pre-heat probe hybridization buffer to 37 °C before use.
5. Re-suspend the pellet with 400 mL of 30 % LMW probe hybridization buffer and pre-hybridize for 30 min at 37 °C.
6. In the meantime, prepare probe solution by adding 2pmol of each probe mixture (odd & even: 1 µL of 2 µM stock per probe mixture) to 100 µL of LMW 30% probe hybridization buffer at 37 °C.
7. Add the probe solution directly to the sample to reach a final probe concentration of 4 nM.
8. Incubate the sample overnight at 37 °C.
9. Pre-heat probe wash buffer to 37 °C before use.
10. Add 1 mL of probe wash buffer to the sample.
11. Centrifuge at RT°C at 4000g for 5 min and remove the wash solution.
12. Re-suspend the cell pellet with 500µL wash solution.
13. Incubate for 5 min at 37°C and remove the wash solution by centrifugation for 5 min.

14. Repeat steps 12 and 13 for two additional times but with 10 min incubation.
15. Proceed to hairpin amplification.

STEP 3: Amplification stage

1. Equilibrate amplification buffer to room temperature before use.
2. Re-suspend the cell pellet with 150 µL of LMW amplification buffer and pre-amplify for 30 min at room temperature.
3. Prepare 15 pmol of each fluorescently labeled hairpin by snap cooling 5 µL of 3 µM stock in hairpin storage buffer (heat at 95 °C for 90 seconds and cool to room temperature in a dark drawer for 30 min).
3. Prepare hairpin mixture by adding all snap-cooled hairpins to 100 µL of LMW amplification buffer at room temperature.
4. Add the hairpin mixture directly to the sample to reach a final hairpin concentration of 60 nM.
5. Incubate the sample for 45 min in the dark at room temperature.
6. Add 1 mL of 5x sodium chloride sodium citrate with 0.1 % Tween 20 (SSCT) buffer at room temperature to the sample to dilute the solution.
7. Centrifuge at room temperature (~25 °C) at 4000 g for 5 min and remove the hairpin solution.
8. Resuspend cells with 500 µL of 5x SSCT buffer with diamidino-2-phenylindole (DAPI) (1 ml of 5 mg/mL stock) and incubate 5 min at room temperature.
9. Centrifuge at room temperature (~25 °C) at 4000 g for 5 min and remove the DAPI solution.
10. Re-suspend the cell pellet with 500 µL of 5x SSCT buffer and incubate 5 min at room temperature.
11. Centrifuge at RT °C at 4000 g for 5 min and remove the wash solution.
12. Repeat steps 10 and 11 for two additional times but with a 10-min incubation.
13. Re-suspend the cell pellet in 50 ml of 5x SSC buffer.
14. Store the sample at 4 °C before imaging.

## Supplementary Note 3: Attribution of performance to sample preparation (antibiotic treatment)

Before measuring fluorescent protein by cytometry, antibiotics are added to freshly harvested cells to halt translation, so that fluorescence measurements reflect levels of gene expression at the time of harvest. Different antibiotics can be used for this purpose, however, differences in measurement that might arise from antibiotic choice are typically not studied.

To compare the effects of antibiotic choice on cytometry measurements of fluorescent protein, we compared two different antibiotic treatments on live cells for flow cytometry measurements of fluorescent protein. We found that kanamycin (Kn, $P_3$) and chloramphenicol (Cm, $P_4$) generally exhibit good agreement in performance, with slight differences in resolvability and relative bias (Supplementary Figs. 12 – 15 and 17). Systematic differences in all four Hill parameters indicated relative bias between the two preparations, although the difference was also typically small. These differences in resolvability and relative bias are presumably due to antibiotic type, although they could also be due to the timing of the measurement, because Cm-treated samples were measured after Kn-treated samples in all three replicates. Across the entire range of induction, Cm-treated samples consistently had slightly better resolvability than samples treated with Kn, although the difference was very small. Like the subtle differences in Hill parameters estimated from Cm-treated and Kn-treated cells, small differences in resolvability between the two antibiotic treatments could also be due to the timing of the measurement.

## Supplementary References

1. Skinner, S. O., Sepúlveda, L. A., Xu, H. & Golding, I. Measuring mRNA copy number in individual Escherichia coli cells using single-molecule fluorescent in situ hybridization. *Nat. Protoc.* **8**, 1100–1113 (2013).
2. So, L. H. *et al.* General properties of transcriptional time series in Escherichia coli. *Nat. Genet.* **43**, 554–560 (2011).
3. Hecht, A., Filliben, J., Munro, S. A. & Salit, M. A minimum information standard for reproducing bench-scale bacterial cell growth and productivity. *Commun. Biol.* **1**, (2018).
4. Nielsen, A. A. K. *et al.* Genetic circuit design automation. *Science (80-. ).* **352**, (2016).
5. Stewart, W. E. Statistics for experimenters, George E. P. Box, William G. Hunter and J. Stuart Hunter, John Wiley & Sons, Inc.(1978), 653 pages. *AIChE J.* (1979). doi:10.1002/aic.690250233
