# Supplementary material for: Comparison of bias and resolvability in single-cell and single-transcript methods
Source: Commun Biol. 2021 Jun 2;4:659. doi: 10.1038/s42003-021-02138-6 (PMC8172639; doi:10.1038/s42003-021-02138-6)
Supplement: Supplementary file 3 — Description of Additional Supplementary Files [file 42003_2021_2138_MOESM3_ESM.pdf]

Description of Additional Supplementary Files

File name: Supplementary data 1
Description: Source data for figures.
