# Supplementary material for: Comparison of bias and resolvability in single-cell and single-transcript methods
Source: Commun Biol. 2021 Jun 2;4:659. doi: 10.1038/s42003-021-02138-6 (PMC8172639; doi:10.1038/s42003-021-02138-6)
Supplement: Supplementary file 1 — Peer Review File [file 42003_2021_2138_MOESM1_ESM.pdf]

Reviewers' comments:

Reviewer #1 (Remarks to the Author):

In this manuscript by Rammohan et al, the authors describe BRASS: Bias and Resolvability Attribution using Split Samples (BRASS), wherein the authors quantitatively evaluate bias and resolvability of single-cell and transcript detection across 12 methods spanning different sample preparation (FISH or HCR), signal detection (Flow cytometry or Microscopy) and measured molecule (RNA, protein or estimated RNA counts). To facilitate effective batch matched comparison, the authors starting from a big batch of cells, split at each measurement step (Sample prep, signal detection and measured molecule). The authors use E.coli cells harboring IPTG inducible eYFP plasmid to measure dosage-dependent activation, alongside positive control (used also for relative promotion unit normalization) and negative control (lacking eYFP plasmid).

The resolvability is defined as the area under the ROC (Receiver operating characteristics) curve (AUC), measured across different IPTG doses. The bias is measured by the fitting the non-linear IPTG dose-response curves to Hills equation, and a non-parametric Friedman test for method comparison. The authors report multiple observations from their comparisons, including (i) higher resolvability of RNA over proteins (using FISH prep) and reverse using HCR, (ii) higher resolvability of microscopy over Flow cytometry over all measurements (iii) increased resolvability of FISH over HCR (for RNA) using Flow cytometry, while comparable using microscopy. Lastly, the authors compare bias and resolvability of FISH against HCR, by converting total RNA fluorescence to single-transcript estimates.
T
he study is systematic, well performed and the data, analysis and interpretation seem robust. There are several caveats in the benchmarking, which the authors also state in manuscript. There are two main comments to the authors

1. While the IPTG dosage to YFP response is a well-established readout of gene regulation, there are several other cellular cues-and-responses occurring at varied timescales and complex regulatory networks. This is particularly true for eukaryotic systems. The authors could highlight other bacterial processes and potentially comment on complex-gene regulatory cascades.
2. The transcriptional burst parameter estimates assessed in this study could be compared to available public data, to better highlight advantages and tradeoffs with such benchmarking.

Reviewer #3 (Remarks to the Author):

Authors demonstrate a sample split method that is followed by multiple measurements that can detect RNA and protein levels.

The paper is important and useful for resolving cell-cell differences, but the following can be addressed to strengthen the paper:

1) HCR flow protein - terminology, needs to be defined better. HCR can be conjugated to antibodies to directly detect proteins. However, it is referred here as something else. As it reads, the manuscript is confusing.

2) The sample split is good idea, but the authors can even use the same samples to quantify FISH and HCR using DNAse treatments similar to sequential FISH or MERFISH strategies. The FISH can be labeled and imaged. And then, HCR can be imaged on the same sample, followed by direct comparisons. Authors should discuss this strategy.

3) For HCR and RNA microscopy, the diffraction limit should be taken into account per single

transcript.

4) What is the effect of sample size distribution on these models?

5) How did the model take into account non-specific binding of labels, especially for HCR.

Point-by-point Response: Comparison of bias and resolvability in single-cell and single-transcript methods

We are grateful for the thoughtful critique of our work by the reviewers and editors, and here we comprehensively address all comments in a point-by-point fashion. We have paid particular attention to points that were emphasized in the Editor's suggestion, "We therefore invite you to revise and resubmit your manuscript, taking into account the points raised. In particular, please carefully address point 2 by reviewer 1 and point 3 and 5 by reviewer 2." We hope that this response and the accompanying revisions will sufficiently address the reviewers' and editor's comments.

For this response, **reviewer comments are shown in bold.** *Manuscript text is shown in italics, with all edits shown in red.* According to guidance from the journal, the corresponding edits in the accompanying Main text document are also highlighted in red, and changes in the accompanying Supplemental Information document are not highlighted.

Reviewer #1

1. **While the IPTG dosage to YFP response is a well-established readout of gene regulation, there are several other cellular cues-and-responses occurring at varied timescales and complex regulatory networks. This is particularly true for eukaryotic systems. The authors could highlight other bacterial processes and potentially comment on complex-gene regulatory cascades.**

We thank the reviewer for recognizing that our approach is potentially applicable to more complex regulatory networks spanning all domains of life. To highlight these potential applications, we have added the following text to the Discussion:

Line 374:
While the focus of this work was to demonstrate a way to compare methods using a model system of bacterial sense and response, BRASS can potentially be used to compare methods for studying more complex systems. For example, single-cell microscopy has been used to analyze how well cells resolve stimulus and transmit information in eukaryotic signaling cascades[37], however the influence of measurement methods on bias and resolvability is typically not addressed in such studies. BRASS could be used to compare experimental methods for analyzing such systems. In the most general sense, we anticipate that a better understanding of bias and resolvability in single-cell methods will directly improve our understanding of complex biological function.

2. **The transcriptional burst parameter estimates assessed in this study could be compared to available public data, to better highlight advantages and tradeoffs with such benchmarking.**

We thank the reviewer for this thoughtful suggestion and have incorporated this comparison accordingly. We initially avoided comparison to available public data, because the focus of this manuscript is to demonstrate a way to compare methods within a single study. However, we recognize that transcriptional burst parameters have been studied previously by a number of groups, so the reviewer's request to understand how our parameters compare to those estimated in previous studies is well-received, especially if the purpose of the comparison is to better highlight advantages and tradeoffs of benchmarking with a split sample approach.

To compare our burst parameters to available public data, and to better highlight advantages and tradeoffs with benchmarking using our approach, we have added the following text to the Results:

Line 335:
Our estimates of burst size and frequency are comparable to previous studies that use the same two-state promoter model to interpret single-molecule RNA FISH measurements of IPTG-induced transcription in E.coli [21,32](Supplemental Figures 21 and 22). Differences between burst parameter estimates can possibly be attributed to differences between experimental conditions used in each study, which underscores the challenge of comparing estimates of biological function between different studies. This challenge of comparability highlights the tradeoffs and advantages of split-sample benchmarking as we have demonstrated here: although performing experiments with HCR and FISH in parallel came at a cost, in a single study we are able to directly compare transcriptional burst parameters between two different RNA labeling strategies performed under the same experimental conditions.

Supplementary Figures 21 and 22 are shown on the following pages.

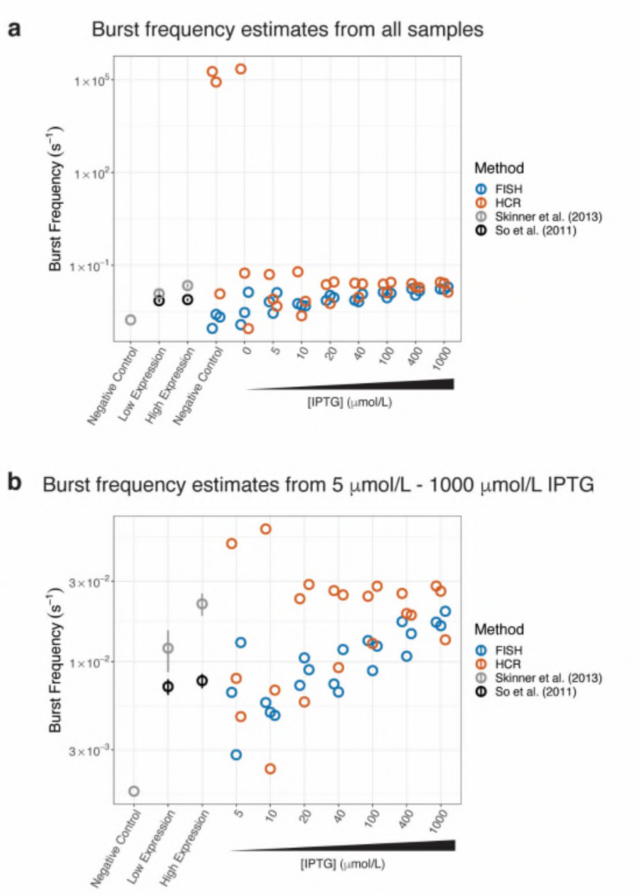

Figure 21. Estimates of burst frequency. (a) Estimates of burst frequency are shown for all samples in this study, for both FISH (blue circles) and HCR (red circles). Also shown are estimates of burst frequency from previous studies using FISH for single-transcript detection in E.coli (gray and black circles, references 7 and 8, respectively). Error bars for data from previous studies represent uncertainty as estimated in references 7 and 8. (b) Same as (a), except that samples in this study are only shown from 5 μmol/L to 1000 μmol/L IPTG for easier comparison.

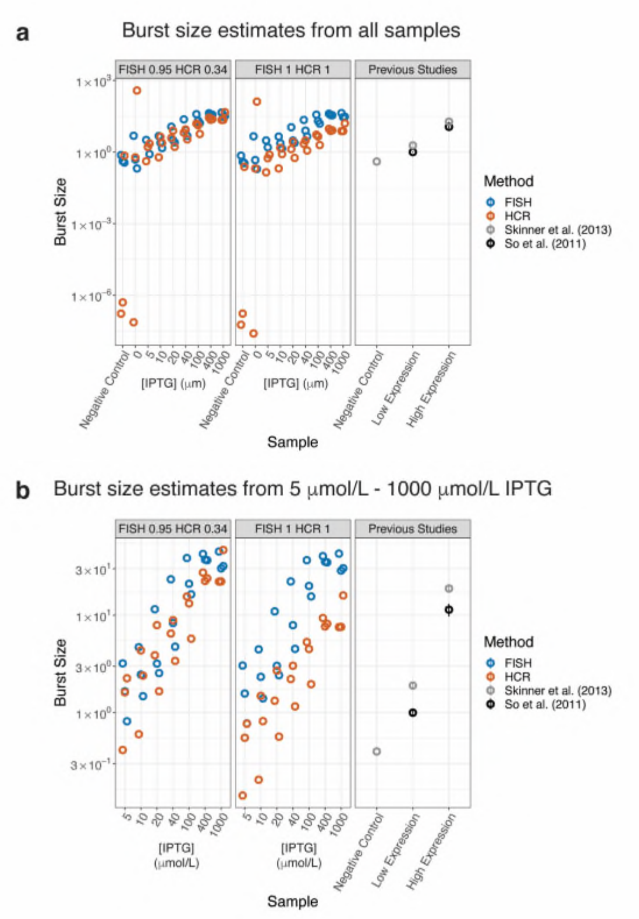

Figure 22. Estimates of burst size. (a) Estimates of burst size are shown for all samples in this study, for both FISH (blue circles) and HCR (red circles). *Left:* Estimates of burst size assuming hybridization efficiencies of 0.95 for FISH and 0.34 for HCR. *Middle:* Estimates of burst size for FISH and HCR without any adjustment for hybridization efficiency. *Right:* Estimates of burst size from previous studies using FISH for single-transcript detection in *E.coli* (gray and black circles)[1,2]. Error bars for data from previous studies represent uncertainty as estimated in references 1 and 2. (b) Same as (a), except that samples in this study are only shown from 5 μmol/L to 1000 μmol/L IPTG for easier comparison.

Reviewer #3

1. **HCR flow protein - terminology, needs to be defined better. HCR can be conjugated to antibodies to directly detect proteins. However, it is referred here as something else. As it reads, the manuscript is confusing.**

We appreciate the reviewer bringing this ambiguity in terminology to our attention. Here, we will comment on our use of "HCR" as well as the phrase "HCR flow protein".

First, we will address our use of the acronym "HCR". We use "HCR" to refer to Hybridization Chain Reaction, which is a method for detecting RNA transcripts using *in situ* hybridization. We establish our definition of "HCR" in the second paragraph of the main text:

Line 55:
…a new method for single-transcript detection in bacteria (hybridization chain reaction, HCR)[20]*.*

Reference 20 (Choi et al., 2018) provides a detailed explanation of HCR, which we also summarize schematically in Supplementary Fig. 2. This definition, reference, and figure explicitly distinguishes our use of "HCR", from antibody-based detection of the HCR protein (for example, using this HCR antibody: https://www.scbt.com/p/hcr-antibody-e-4).

We use a table and consistent set of symbols in Figures 1a and 1b to establish that "HCR flow protein" refers to Sample Preparation, Signal Detection, and Measurand, and those symbols are also used consistently in Figs. 2-5. However, based on the reviewer's comment, we understand that the definition of "HCR flow protein" may not be clear.

To clarify our uses of "HCR" and "HCR flow protein" in this work, we have edited the main text as follows:

Line 90:
Each of the 12 methods can be described by a unique sequence of measurement steps for sample preparation, signal detection, and measurand. We used several different sample preparations, including two different antibiotic treatments to halt translation prior to flow cytometry detection of fluorescent protein (kanamycin, Kn, and chloramphenicol, Cm), and two different techniques for labeling RNA via in situ hybridization (FISH and HCR) (Supplementary Fig. 2). After sample preparation, we split samples between two different signal detection methods: flow cytometry (abbreviated "flow" in Figs. 1-5), and microscopy. Finally, we chose three different measurands for the level of gene expression per cell: whole-cell fluorescence from protein, whole-cell fluorescence from labeled RNA, and estimated RNA count per cell ("RNA #" in Fig. 1a, Supplementary Fig. 5). We refer to methods according to their unique combination of sample preparation, signal detection, and measurand. For example, "HCR flow protein" refers to a method consisting of hybridization chain reaction (sample preparation), flow cytometry (signal detection), and protein (measurand).

Line 134:
Flow cytometry measurement of fluorescent protein expression levels following RNA-labeling by HCR ("HCR Flow Protein" in Fig. 1) has a similar AUC profile maximum of ≈0.90 (Fig. 1b).

2. **The sample split is good idea, but the authors can even use the same samples to quantify FISH and HCR using DNAse treatments similar to sequential FISH or MERFISH strategies. The FISH can be labeled and imaged. And then, HCR can be imaged on the same sample, followed by direct comparisons. Authors should discuss this strategy.**

We agree with the reviewer that sequential labeling is an alternative strategy to split samples for directly comparing FISH and HCR on the same sample. Additionally, simultaneous labeling by FISH and HCR within the same cells can be performed. However, both of these strategies of method comparison require assumptions that are avoided by a split sample approach.

To acknowledge and discuss these possible alternatives to a split sample approach, we have included the following statement in the main text (Discussion):

Line 365
As an alternative approach to split samples for comparing RNA labeling strategies in a single study, the same cells could be labeled by both FISH and HCR. This could potentially be done using sequential labeling of the same sample, utilizing DNAse treatment to strip one label before another label is used for analysis[34]. Or, direct comparison of FISH to HCR could be achieved using simultaneous labeling by designing probes that hybridize to different regions of the target mRNA[35–37]. However, these kinds of method comparisons assume that DNAse treatment or mRNA target sequence do not affect method performance, whereas a split sample comparison does not require such assumptions.

3. **For HCR and RNA microscopy, the diffraction limit should be taken into account per single transcript.**

When analyzing microscopy of RNA transcripts labeled by either HCR or FISH, we account for the diffraction limit when detecting fluorescent spots within the cells using analysis strategies that are well-established and widely-accepted within the field. The protocols are referenced in the Supplemental Information sub-section titled "Microscopy image analysis" (pages 36-37). We understand that the explanation we provided in our initial draft did not explicitly address the diffraction limit, and we appreciate the reviewer bringing this to our attention.

To clarify how the diffraction limit is accounted for in our single-transcript analysis, we have edited steps 4 and 9 of the "Microscopy image analysis" section in Supplemental Information:

Page 36:
4. Spot detection was performed as previously described using Spatzcells[7]. This program quantifies the location and fluorescence intensity of diffraction-limited spots in the following manner. Gaussian smoothing is applied to reduce the contribution of pixel-to-pixel noise. 2D

local maxima are detected in each z-slice, and then maxima are matched between z-slices. The locations of these maxima are fit to 2D Gaussian functions.

Page 37:
9. Because spots cannot always be spatially-separated from one another in diffraction-limited microscopy, we use a calibration method to estimate the number of spots per cell area based on the integrated brightness of spots per cell area, after correcting for background subtraction. This method is described in detail in reference 1. In this manner, we are able to use integrated spot brightness to account for spots that cannot be spatially-separated in diffraction-limited microscopy.

4. **What is the effect of sample size distribution on these models?**

We thank the reviewer for this thoughtful question. It is not immediately apparent what is meant by the phrase "sample size distribution", which could refer to the number of cells (in this study, typically 10^2 – 10^5) in a measured distribution for a given sample; or, the variation (distribution) in the number of measured cells among different methods; or, the variation (distribution) of sample sizes among replicates.

In general, assuming individual cells are independent of one another within a given population, a larger sample size is a better representation of the population of interest. In terms of the estimates of our model parameters, we would expect larger samples would lead to improved estimates with lower uncertainties. These statistical effects are widely-known. We considered adding additional text to this effect in the main text, but realized we haven't made any novel observations about these concepts, and so we have not edited our text with regard to this point.

Assuming the reviewer is referring to the number of cells that make up a measured distribution, we considered that the most marked difference in sample sizes between methods occurs when comparing microscopy (typical measurement uses 10^2 – 10^3 cells) to flow cytometry (typical measurement uses 10^5 – 10^6 cells). This difference in "sample size" between methods may influence the reproducibility of our resolvability assessment, however, we generally do not observe evidence of bias in Hill parameters between microscopy and flow cytometry.

These observations are already included in the main text:

Line 314:
Replicate-to-replicate variability of resolvability was higher for microscopy than flow cytometry. One possible explanation for microscopy's higher variability is lower throughput (microscopy typically measures 10^2 cells – 10^3 cells, whereas flow cytometry typically measures 10^5 – 10^6 cells), so quantitative differences between microscopy distributions may be less reproducible.

Signal detection methods generally do not exhibit relative bias for parameterization of dose-response (Fig. 2 and Supplementary Figs. 12 – 15).

5. **How did the model take into account non-specific binding of labels, especially for HCR.**

We agree with the reviewer that accounting for non-specific binding of labels is important, for both HCR and FISH. Importantly, for all methods, we measured non-specific signal using a negative control strain of *E. coli* lacking the target *eyfp* transcript (pAN1201). Because these cells did not contain the target transcript, we attributed any FISH or HCR signal measured in pAN1201 to non-specific binding of labels.

We used 2 different models to analyze cellular response. We analyze dose-response using the Hill function model, and we analyze transcriptional burst kinetics using a two-state promoter model. Here, we will address how both models take into account non-specific binding of labels.

First, we will address how non-specific signal is taken into account when modeling dose-response. For analyzing dose-response in Relative Promoter Units, the non-specific signal from pAN1201 is subtracted from pAN1717 and pAN1818 before fitting to a Hill function. This is described in the Method sub-section titled "Normalization to Relative Promoter Units (RPU)". To more explicitly describe how this model accounts for non-specific signal, we have added text to that section of the Methods:

Line 559:
Normalization to Relative Promoter Units (RPU) was performed in the following manner, where "Signal" indicates the median of a measured distribution of the indicated sample:

$$RPU = \frac{Signal_{pAN1818} - Signal_{pAN1201}}{Signal_{pAN1717} - Signal_{pAN1201}}$$

Here, pAN1818 is a sample used to measure inducible gene expression, pAN1717 is a sample used to measure constitutive gene expression from the J23101 promoter, and pAN1201 is a sample lacking the expression system (used as a negative control to measure non-specific signal).

Next, we will discuss how we account for non-specific signal in our modeling of transcriptional bursting. This model relies on estimates of number of transcripts per cell from microscopy images, and does not include any explicit terms for non-specific signal. Instead, prior to modeling, we account for non-specific binding of labels for FISH and HCR in our analysis of the raw data, as described in the Supplemental Information sub-section titled "Microscopy Image Analysis". Specifically, non-specific signal is explicitly addressed in steps 7 and 8:

Page 36:

7. *Because the RNA transcript was relatively short (720 nt), there was overlap in spot brightness distributions for non-specific and specific signal. A mixture of 2 lognormal distributions constrained to be centered on either side of a threshold intensity was used*

to fit the spot intensity histograms, to estimate the total spot intensity due to specific signal.

8. *Artifact exclusion for microscopy was necessary because non-specific fluorescence outside of cell bodies were occasionally in close enough proximity to the cells to affect the fluorescence within the segmented cell. Cells were visually inspected to exclude the influence of non-specific fluorescence on image analysis. Some cells were located near non-specific fluorescence that originated outside of the cell body. These cells were excluded from analysis. Cells for which fluorescence originated from spots inside of the cell body were included in the analysis.*

To further consider the role of non-specific signal in our modeling of transcriptional bursting, we applied our model of transcriptional bursting to the negative control (pAN1201) samples, which only contained non-specific signal. We found that the negative binomial distribution goodness-of-fit in the negative controls was comparable to other samples containing the target mRNA transcript, and we estimated burst size and frequency parameters from the negative controls. These estimates are included in new plots (Supplementary Figures 21 and 22), and the following text has been added to the Methods section titled "Parameterization of transcriptional bursting using negative binomial distributions":

Line 648:
To assess the role of non-specific signal in our modeling of transcriptional bursting, we applied our model of transcriptional bursting to our negative control samples. For both FISH and HCR, estimates of burst size and frequency from negative controls were similar to those from the lowest expression samples (0 IPTG) (Supplementary Figures 21 and 22).

Other changes

1. We have added a section titled "Statistics and Reproducibility" to the Methods section:

Line 655:
Statistics and reproducibility
The names of all statistical tests and P values are noted in the text. In general, we provide two-sided p-values for sign tests, except for P values for Friedman tests, which are inherently one-sided. We do not rely on alpha values for drawing conclusions. The number of cells in every measurement is provided in Supplemental Table 4. The median was used for analyses that required estimating the central tendency of single-cell distributions. Uncertainty on the median was estimated using bootstrapping with 1,000 iterations. Uncertainty on Hill parameters was estimated using 95 % confidence intervals of nonlinear least-squares fit.

A total of three biological replicates were performed on different days. To clearly present replicate-to-replicate variability, results from each replicate are plotted separately in every figure.

As a general approach to processing, the inferences in this work treat the observations that remain after processing as a random sample of the population for characterization, which in this

case is the entire population of E. coli cells growing in culture. We assume the filtering that has been done eliminates experimental artifacts and does not suppress or accentuate subsets of the general E. coli population. We do not incorporate any uncertainty due to potential biases, which if present, could dramatically increase uncertainty characterizations.

2. We have made the results of single-cell and single-transcript measurements publicly available, and have included a link to the data in the section titled "Data availability":

Line 681:
Source data for figures is available through the NIST Data Science Portal (https://doi.org/10.18434/mds2-2300) and Supplementary Data file.

3. According to general guidance from the journal regarding use of the word "new", claims of novelty with regard to single-transcript detection by HCR have been removed.
4. We changed all instances of "4000 *g*" to "4000 x *g*" (Methods).
5. We changed all instances of "paired sign t-test" to "two-sided p-value of a sign test" (Captions for Main Figs. 3-6, Supplemental Figs. 17-19)
6. The title of Figure 6g in the Main Text was changed from "Burst size w/ detection efficiency" to "Burst size w/ hybridization efficiency", to match the description of "hybridization efficiency" in the text. Additionally, the caption was modified for clarity (updated version shown on next page).
7. Minor adjustments were made to Supplemental Figure 2.
8. Other minor typographical corrections are shown in red in the Main text.

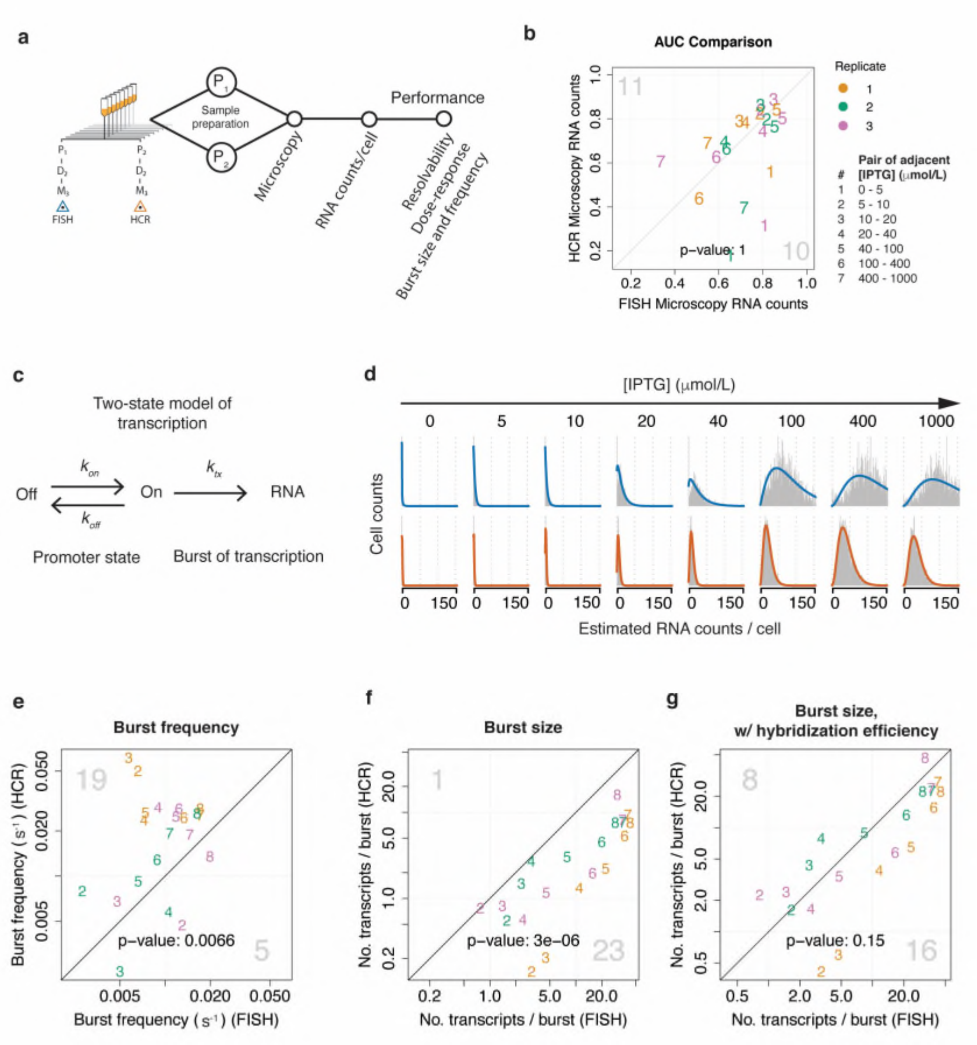

Figure 1. Performance of single-transcript methods can be attributed to RNA labeling strategy. (a) Performance of single-transcript methods was compared using cells that shared the same steps of the measurement process except for the RNA labeling step. (b) Resolvability of FISH and HCR was assessed by plotting AUC calculated from adjacent stimulus levels. Pairs of adjacent IPTG concentrations are shown as numbers within the plots, as indicated in figure legend. (c) A two-state promoter model was used to evaluate transcription kinetics. (d) Negative binomials were used to fit single-transcript distributions for FISH (blue) and HCR (dark orange). (e) Estimates of burst frequency are plotted for FISH versus HCR. The RNA lifetime was assumed to be a constant (2.8 minutes). (f) Estimates of burst size, and (g) estimates of burst size after correcting for hybridization efficiency, are plotted for FISH versus HCR. For parts f and g, burst size is the number of transcripts per burst. For parts e, f, and g, the scatter plot numbers 2, 3, 4, 5, 6, 7, and 8 represent 5 μmol/L, 10 μmol/L, 20 μmol/L, 40 μmol/L, 100 μmol/L, 400 μmol/L, and 1000 μmol/L IPTG, respectively. The 0 μmol/L IPTG case is not shown, in order to more easily see the trend for the remaining induction conditions. For (b), (e), (f), and (g): Diagonal line indicates equivalent performance between the two methods. Color indicates biological replicates one (orange), two (green), and three (purple), as indicated in figure legend. Large gray numbers in the top-left and bottom right-corners indicate how many values were higher for the method plotted on the y-axis or x-axis, respectively. The two-sided p-values for a sign test are shown within each plot.

This concludes our point-by-point response to the reviewers and editors. We are hopeful that the discussion provided here, along with the changes that we have implemented in our manuscript, sufficiently address any concerns the editors may have about accepting this manuscript for publication.

Sincerely,

Jayan Rammohan
Research Chemist, Cellular Engineering Group
National Institute of Standards and Technology

David Ross
Project Leader, Cellular Engineering Group
National Institute of Standards and Technology

REVIEWERS' COMMENTS:

Reviewer #1 (Remarks to the Author):

The authors have address my comments, and incorporated the raised points. I would recommend publication of ms.

Reviewer #3 (Remarks to the Author):

The authors have address the concerns, thanks for providing insights into joint discussions. This type of standardization effort will help enhance the reproducibility in single-cell genomics field. The paper is suitable for publication.
